# Conditional *Myh9* and *Myh10* inactivation in adult mouse renal epithelium results in progressive kidney disease

**DOI:** 10.1172/jci.insight.138530

**Published:** 2020-11-05

**Authors:** Karla L. Otterpohl, Brook W. Busselman, Ishara Ratnayake, Ryan G. Hart, Kimberly R. Hart, Claire M. Evans, Carrie L. Phillips, Jordan R. Beach, Phil Ahrenkiel, Bruce A. Molitoris, Kameswaran Surendran, Indra Chandrasekar

**Affiliations:** 1 Enabling Technologies Group, Sanford Research, Sioux Falls, South Dakota, USA.; 2 Basic Biomedical Sciences Graduate Program, University of South Dakota, Vermillion, South Dakota, USA.; 3 Department of Nanoscience and Nanoengineering, South Dakota School of Mines and Technology, Rapid City, South Dakota, USA.; 4 Department of Pediatrics, University of South Dakota Sanford School of Medicine, Sioux Falls, South Dakota, USA.; 5 Histology and Imaging Core, Sanford Research, Sioux Falls, South Dakota, USA.; 6 Department of Pathology and Laboratory Medicine, Indiana University School of Medicine, Indianapolis, Indiana, USA.; 7 Department of Cell and Molecular Physiology, Loyola University Chicago Stritch School of Medicine, Maywood, Illinois, USA.; 8 Department of Medicine, Indiana University School of Medicine, Indianapolis, Indiana, USA.; 9 Pediatrics and Rare Diseases Group, Sanford Research, Sioux Falls, South Dakota, USA.

**Keywords:** Cell Biology, Nephrology, Chronic kidney disease, Cytoskeleton, Molecular genetics

## Abstract

Actin-associated nonmuscle myosin II (NM2) motor proteins play critical roles in a myriad of cellular functions, including endocytosis and organelle transport pathways. Cell type–specific expression and unique subcellular localization of the NM2 proteins, encoded by the *Myh9* and *Myh10* genes, in the mouse kidney tubules led us to hypothesize that these proteins have specialized functional roles within the renal epithelium. Inducible conditional knockout (cKO) of *Myh9* and *Myh10* in the renal tubules of adult mice resulted in progressive kidney disease. Prior to overt renal tubular injury, we observed intracellular accumulation of the glycosylphosphatidylinositol-anchored protein uromodulin (UMOD) and gradual loss of Na^+^ K^+^ 2Cl^–^ cotransporter from the apical membrane of the thick ascending limb epithelia. The UMOD accumulation coincided with expansion of endoplasmic reticulum (ER) tubules and activation of ER stress and unfolded protein response pathways in *Myh9&10*-cKO kidneys. We conclude that NM2 proteins are required for localization and transport of UMOD and loss of function results in accumulation of UMOD and ER stress–mediated progressive renal tubulointerstitial disease. These observations establish cell type–specific role(s) for NM2 proteins in regulation of specialized renal epithelial transport pathways and reveal the possibility that human kidney disease associated with *MYH9* mutations could be of renal epithelial origin.

## Abstract 2

Adult mouse renal epithelium specific knockout of Myh9 and Myh10 genes result in kidney disease.

## Graphical abstract

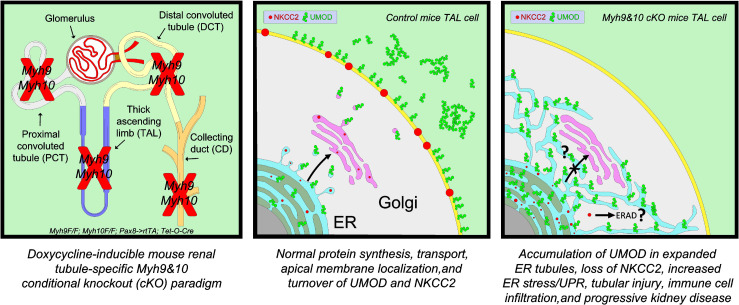

## Introduction

The diverse epithelial cells that populate the tubular segments of the kidney express unique membrane proteins that regulate solute, ion, and pH homeostasis (1–3). The transport machinery that facilitates apical versus basolateral sorting of receptors, channels, and cotransporters in these polarized epithelia are complex and involve coordination of signals from hormones and other molecules (2, 4, 5). The actin cytoskeleton and associated myosin motors play essential roles in membrane protein trafficking, including endocytosis, anterograde and post-Golgi transport, and transcytosis, in epithelial cells (6–10). For example, in renal proximal tubules, myosin VI drives the sodium hydrogen exchanger (NHE3) and sodium phosphate cotransporter to the brush borders (11). Additionally, a role for actin cytoskeleton–mediated vesicular transport of the aquaporin-2 water channel to the apical membrane of collecting duct principal cells has been described (12–14). Recent studies have shown that nonmuscle myosin II (NM2) proteins are also involved in RAB6-mediated trans-Golgi network fission (8) and exocytosis of giant vesicles in the salivary gland (9).

To add to this emerging scientific evidence, our previous work identified critical roles for NM2 proteins, NM2A and NM2B, encoded by the paralogous genes *Myh9* and *Myh10*, respectively, in membrane remodeling events during mammalian endocytosis (6, 7). Our recent work also demonstrated that the NM2 proteins have overlapping as well as cell type–specific expression and membrane localization pattern in murine renal tubular segments (15). Taken together, we hypothesized that the NM2 proteins have unique, cell type–specific role(s) in regulating the renal epithelial transport machinery. In order to determine the role(s) for *Myh9* and *Myh10* in the renal epithelial cell types, we generated a conditional knockout (cKO) mouse model using *Pax8*→*rtTA Tet-O-Cre* to facilitate doxycycline-inducible (16) adult renal epithelium–specific loss of MYH9&10 proteins, while leaving their glomerular expression intact. Loss of NM2 proteins from renal epithelial segments resulted in rapidly progressing kidney disease and revealed critical roles for NM2 proteins in regulation of unique, thick ascending limb–associated (TAL-associated) proteins uromodulin (UMOD) and Na^+^ K^+^ 2Cl^–^ cotransporter (NKCC2). Ultrastructural and immunohistologic analysis of *Myh9&10*-cKO kidneys identified an expansion of the endoplasmic reticulum (ER) tubules associated with UMOD accumulation within the TAL cells. Progressive activation of the ER stress and unfolded protein response pathway as well as alterations in ER chaperone localization and expression were also observed in the cKO kidneys. Our results reveal a renal tubule–specific role for NM2 proteins and provide insights into the specialized renal epithelial transport mediated by NM2 molecular motors.

## Results

### Conditional genetic inactivation of Myh9 (NM2A) and Myh10 (NM2B) in adult mouse renal epithelia results in progressive kidney disease.

MYH9&10 proteins have a partially overlapping expression pattern in murine tubular epithelial segments (15). In order to uncover redundant functions of NM2 proteins in the kidney epithelial compartments, as well as to model a severe loss-of-function phenotype, we inactivated both alleles of *Myh9* and *Myh10* only in the adult renal epithelium. Doxycycline treatment of cohorts of *Myh9&10*-cKO and control littermate mice was initiated at 4 weeks of age, and kidney structure and function were evaluated at 4, 6, 9, and 12 weeks of age. We confirmed the loss of MYH9 and MYH10 protein expression in the renal tubular segments of cKO mice at 6 weeks (Supplemental Figures 1 and 2; supplemental material available online with this article; https://doi.org/10.1172/jci.insight.138530DS1).

**Figure 1:**
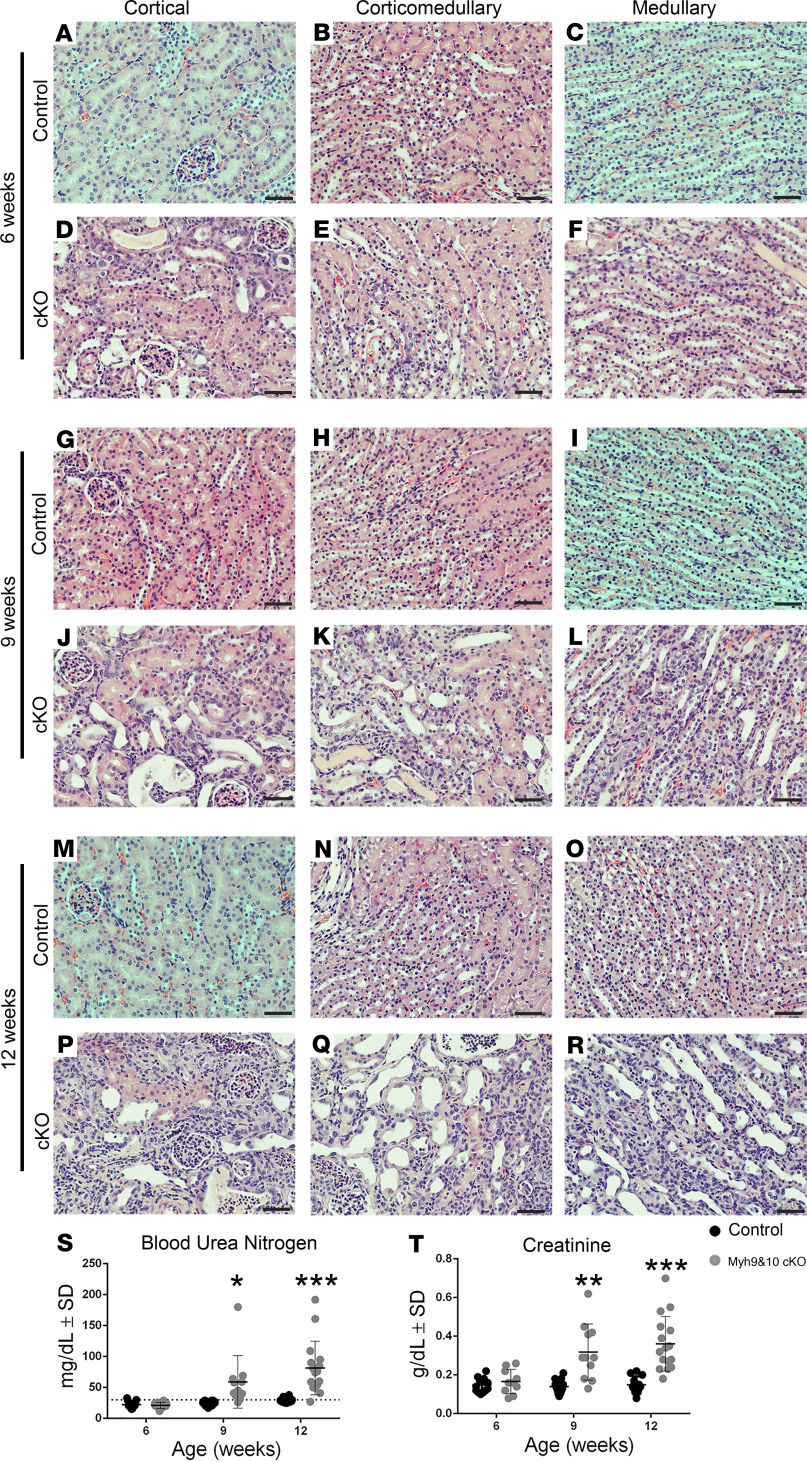
Conditional genetic inactivation of *Myh9* and *Myh10* in adult mouse renal tubular segments results in progressive kidney disease. (**A**–**R**) Kidney sections from *Myh9&10*-cKO mice and control littermates stained with H&E (*n* > 3 for controls and cKO at each time point). Normal histology was observed in control kidneys at all time points (6 weeks: **A**–**C**, 9 weeks: **G**–**I**, and 12 weeks: **M**–**O**). (**D**–**F**) Minor tubular dilation is observed in the *Myh9&10*-cKO mice at 6 weeks in the cortical region (**D**), while the corticomedullary (**E**) and medullary (**F**) regions appeared normal. (**J**–**L**) Tubular dilation is observed in the cortical, corticomedullary and medullary regions at 9 weeks in the cKO mice along with focal cellular infiltration in the interstitium (**J**–**L**). (**P**–**R**) At 12 weeks of age, tubular dilation was increased, and interstitial hypercellularity was detected in all regions. (**S** and **T**) Measurement of blood urea nitrogen (BUN, **S**) and serum creatinine (**T**) indicated decline in kidney function starting at 9 weeks of age. Sample numbers for BUN and serum creatinine for 6 weeks *n* = 13 and 10; 9 weeks *n* = 16 and 12; 12 weeks *n* = 12 and 16 for controls and cKO animals, respectively. Scale bars: 50 μm. *P ≤ 0.05, **P ≤ 0.001, and ***P ≤ 0.0008. The exact *P* values are listed in Supplemental Table 3. SAS software suite developed for multivariate analysis was used for statistical analysis.

Histological analysis of kidney tissues from all time points (ages) with H&E, periodic acid–Schiff (PAS), and Masson’s trichrome staining revealed a progressive increase in tubular injury and interstitial cellular infiltration. At 6 weeks of age, 2 weeks after initiation of *Myh9&10* inactivation, focal regions of dilated tubules in the cortex were observed in the *Myh9&10*-cKO mouse kidneys (Figure 1, D–F, Supplemental Figure 3) compared with littermate controls (Figure 1, A–C). Regions of dilation were expanded at 9 weeks of age (Figure 1, J–L, Supplemental Figure 3) and became more severe by 12 weeks (Figure 1, P–R, Supplemental Figure 3) compared with littermate controls (Figure 1, G–I and M–O, respectively; and Supplemental Figure 3). At 12 weeks we observed renal atrophy with multiple cysts and increased cellular infiltration (Supplemental Figure 3). PAS staining supported the progressive tubular dilation seen in *Myh9&10*-cKO kidneys and showed changes to the brush borders (Supplemental Figure 4). Masson’s trichrome–stained kidney sections showed a few focal regions of the kidney with mild fibrosis in cKO animals (Supplemental Figure 5). Progressive tubular dilation was accompanied by focal interstitial hypercellularity at both the 9- and 12-week time points (Figure 1, J–L and P–R) because of the accumulation of infiltrating cells. We did not observe any significant differences in total protein, pH, or osmolality of the urine between control and cKO cohorts at 4 or 6 weeks of age (Supplemental Figure 6, Supplemental Tables 1 and 2). However, *Myh9&10*-cKO mice had urinary pH levels significantly more acidic than littermate controls at 9- and 12-week time points (Supplemental Figure 6). Analysis of serum chemistry demonstrates a progressive decline in kidney function in the *Myh9&10*-cKO mice compared with the control littermates. *Myh9&10*-cKO mice had significantly higher blood urea nitrogen (BUN) and serum creatinine levels than the control littermates at 9 weeks, and both were further elevated at 12 weeks (Figure 1, S and T; Supplemental Table 3). *Myh9&10*-cKO mice were also smaller and had lower blood glucose levels at 12 weeks (Supplemental Tables 1–3). Glycosuria was apparent in male *Myh9&10*-cKO mice starting at 9 weeks of age (Supplemental Table 2).

### Elevated levels of tubular injury markers and immune cell infiltration highlight disease progression in Myh9&10-cKO mice.

**Figure 2:**
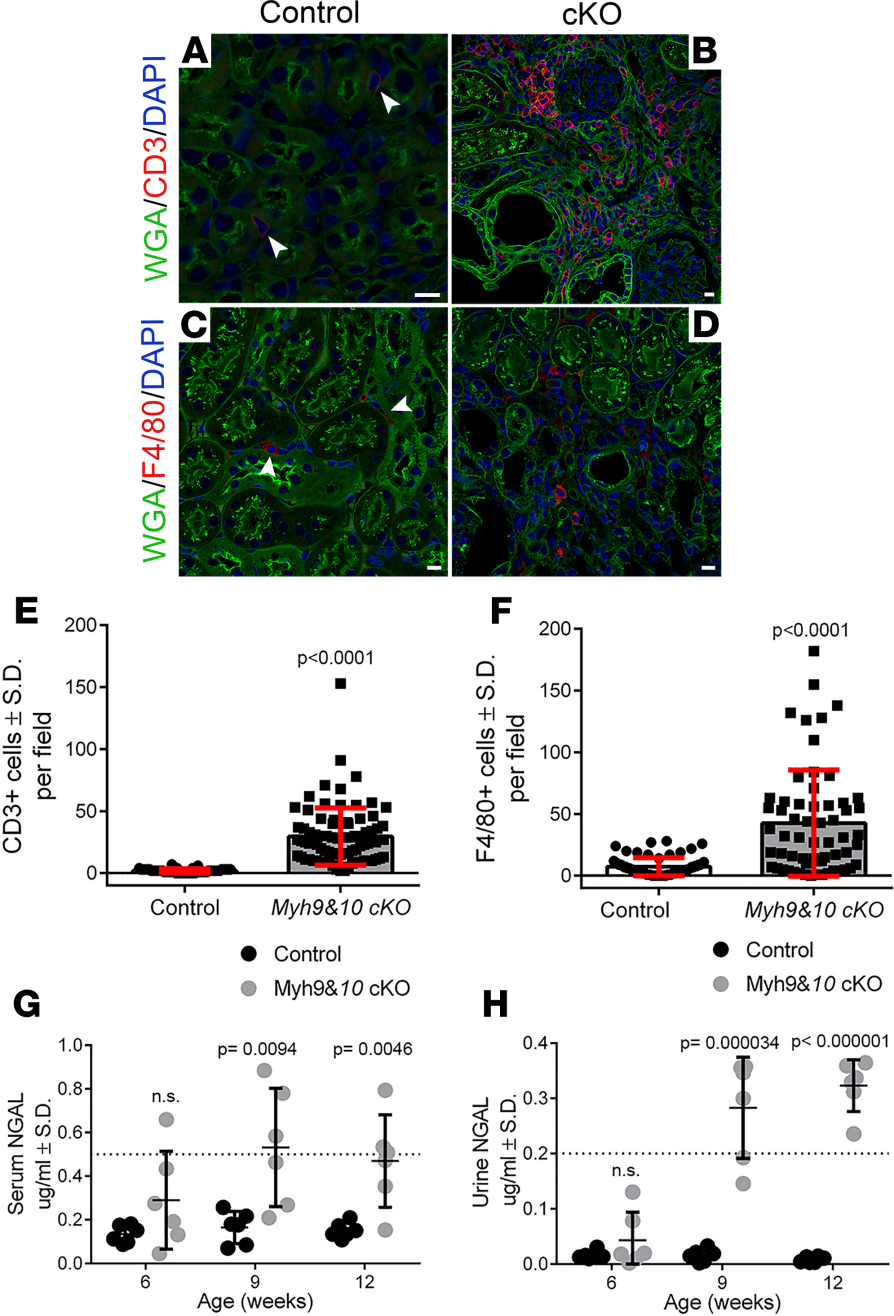
Tubular injury markers indicate tubulointerstitial disease in *Myh9&10*-cKO kidneys. (**A**–**D**) Images from control and cKO kidney sections of 9-week-old cohorts stained for T cell marker CD3 or mouse macrophage marker F4/80. (**A** and **B**) The representative control section shows 2 CD3^+^ (red) cells (**A**, arrowheads) while the cKO section shows several CD3^+^ cells (**B**). (**C** and **D**) The section from control kidney shows F4/80^+^ (red) macrophages (**C**, arrowheads); cKO kidney section shows several F4/80^+^ macrophages (**D**). Scale bar: 10 μm. (**E** and **F**) Graphs show cell counts for CD3^+^ and F4/80^+^ cells in 9-week-old control and cKO kidneys. (**E**) cKO kidneys had increased number of CD3^+^ T cells compared with control kidneys (*P* < 0.0001, *n* = 81 images from 4 control kidneys and 82 images from 4 cKO kidneys). (**F**) F4/80^+^ macrophages were more abundant in the cKO kidneys compared with control kidneys (*P* < 0.0001, *n* = 64 images from 3 control kidneys and 60 images from 3 cKO kidneys). Statistics were done using the unpaired 2-tailed *t* test. (**G** and **H**) NGAL concentrations in the serum and urine of control and cKO mice were determined for the 6-, 9-, and 12-week time points. (**G**) NGAL was significantly elevated in the serum of 9- and 12-week-old cKO mice compared with controls (*P* = 0.0094 and *P* = 0.0046, respectively). (**H**) Urinary NGAL was also significantly elevated in the 9- and 12-week-old cKO mice (*P* = 0.000034 and *P* < 0.000001, respectively). Dotted lines indicate the highest value on the standard curve relative to sample dilution. *n* = 6 controls and 6 cKO samples per time point. Statistics were done using the multiple *t* test, 2-tailed.

To further understand the disease progression in the *Myh9&10*-cKO mouse model, we performed immunostaining of kidney sections to identify the infiltrating cells in *Myh9&10*-cKO kidneys. These cells were determined to be CD3^+^ T cells and F4/80^+^ macrophages (Figure 2, A–D). Quantification of cell numbers from multiple kidney sections showed statistically significant increases in T cells and macrophages in *Myh9&10*-cKO kidneys compared with control sections, confirming an inflammatory response surrounding the damaged tubules (Figure 2, E and F). This prompted us to investigate whether tubular injury markers were also elevated in response to the progressive tubular damage. Neutrophil gelatinase-associated lipocalin (NGAL) is a small circulating protein biomarker that is elevated in both serum and urine during acute kidney injury (AKI) and chronic kidney disease (CKD) (17). NGAL is known to play a protective role in AKI; however, prolonged exposure is considered harmful due to its proinflammatory effects, which promote progression of CKD (18). ELISA analysis of serum and urine samples detected high NGAL levels at both 9-week and 12-week time points in *Myh9&10*-cKO samples compared with the controls. The NGAL values (μg/mL) also demonstrated an increase from 6 to 12 weeks of age in the *Myh9&10*-cKO animals, revealing the progressive nature of the tubular damage (Figure 2, G and H). Because we observed loss of brush borders in the proximal tubules (Supplemental Figure 4), we probed for the presence of kidney injury molecule 1 (KIM1) (19) in urine samples from 9-week control and *Myh9&10*-cKO animals. Immunoblot analysis detected KIM1 protein with molecular weight ~60–70 kDa, in *Myh9&10*-cKO urine samples, while no corresponding protein bands were detected in the control urine, thereby supporting elevated levels of KIM1 in cKO animals (Supplemental Figure 7). Next, we carefully analyzed individual tubular segment–specific phenotypes in *Myh9&10*-cKO mice.

### Myh9&10-cKO mouse proximal tubular segments show minor changes in brush border morphology, but expression of receptor and cotransporters is mostly unaffected.

Elevated levels of KIM1 indicated that the proximal convoluted tubules (PCTs) sustained injury from the loss of MYH9 and MYH10 in the cKO mice. PCTs are major sites of low–molecular weight protein endocytosis/transcytosis through unique LDL family of receptors, such as megalin and cubilin (20). Because our previous work demonstrated a crucial role for NM2 isoforms in receptor-mediated endocytosis (7), we assessed the localization of the PCT receptor, megalin, in 9-week-old kidney sections from cKO and control mice. No changes were observed in the localization and expression pattern of megalin along the apical membrane of proximal tubules in the cKO kidneys (Supplemental Figure 8, D–F) compared with littermate controls (Supplemental Figure 8, A–C).

**Figure 3:**
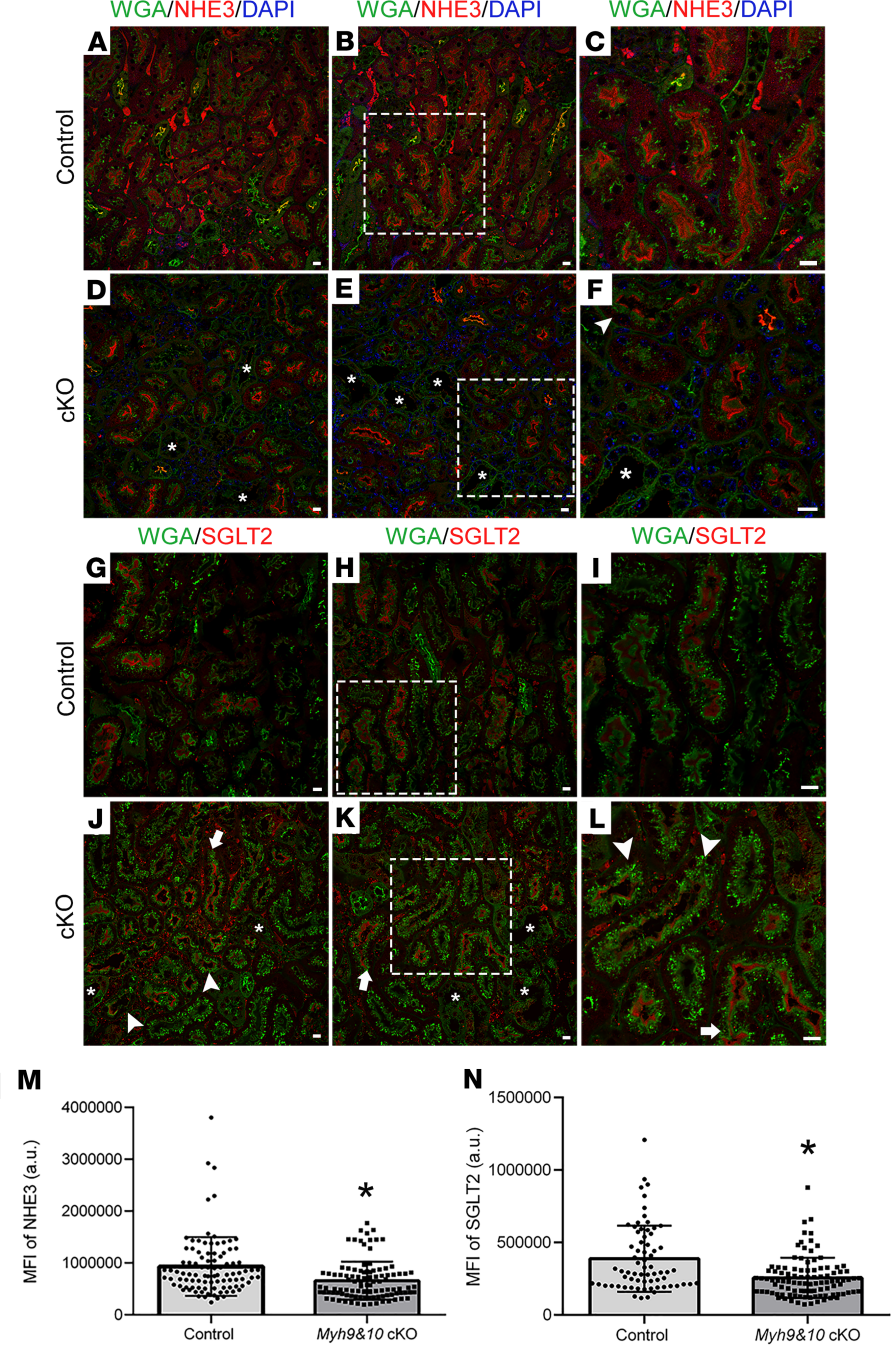
Loss of MYH9 and MYH10 does not affect the localization of proximal tubule–associated sodium cotransporters NHE3 and SGLT2. (**A**–**F**) Bouin’s-fixed kidney sections from 9-week-old cKO mice and control littermates were stained using NHE3 antibody along with Oregon green–488–wheat germ agglutinin (WGA) and DAPI. (**A**–**C**) NHE3 (red) localizes to the PCT brush borders and does not colocalize with WGA (**A** and **B**); intracellular NHE3 staining was also observed. (**D**–**F**) NHE3 staining was reduced in cKO mouse kidney sections, and partial loss of expression along the apical membrane was observed in some tubules with loss of brush border (**F**, white arrowhead). Adjacent tubular segments that are severely dilated are denoted with asterisks (**D**–**F**). (**G**–**L**) Kidney sections from 9-week-old cKO mice and control littermates were stained using an SGLT2 antibody and WGA. (**G**–**I**) Control kidney sections show positive staining for SGLT2 (red) along the brush borders. (**J**–**L**) In the cKO kidneys, SGLT2 expression varied between tubules. Some tubules showed decreased expression (white arrowhead), while other tubules maintained SGLT2 expression (white arrows). Asterisks (*) mark adjacent tubular segments that are severely dilated (**J** and **K**). The white dotted squares (**B**, **E**, **H**, and **K**) denote regions enlarged in **C**, **F**, **I**, and **L**. Scale bar: 10 μm. Images are representative of *n* ≥ 3 kidneys for control and cKO samples. (**M** and **N**) Graphs represent MFI for NHE3 (**M**) and SGLT2 (**N**) that show statistically significant reduction in staining intensity in cKO mice. Control tubules (*n* = 96 for NHE3 and *n* = 69 for SGLT2), cKO tubules (*n* = 108 for NHE3 and *n* = 102 for SGLT2). Error bars show standard deviation of samples. *P < 0.0001, calculated by unpaired 2-tailed *t* test.

Myosin motors also contribute to the regulation of sodium cotransporters in the proximal tubules. Both myosin VI and myosin II play roles in the redistribution of NHE3 to and from the microvilli in response to angiotensin II (21). Therefore, we evaluated the localization of the 2 main PCT-specific sodium cotransporters, NHE3 and sodium glucose cotransporter 2 (SGLT2), along with an unbiased membrane marker (wheat germ agglutinin, WGA). In 9-week-old kidney sections from control mice, we observed NHE3 localization to the brush borders (Figure 3, A–C), whereas *Myh9&10*-cKO kidney sections showed a decrease in NHE3 staining within some PCTs (Figure 3, D–F). Costaining for brush border–associated protein villin along with NHE3 confirmed a loss of brush borders in the PCTs with reduced NHE3 staining in the cKO samples (Supplemental Figure 9). Expression of SGLT2 in *Myh9&10*-cKO kidneys varied, with some tubules showing a pronounced loss while other dilated tubules maintained SGLT2 expression (Figure 3, J–L) similar to control kidneys (Figure 3, G–I). Fluorescence intensity measurements along multiple PCTs from at least 3 mice showed a small but significant decrease in NHE3 and SGLT2 protein expression along the apical membrane in cKO mice (Figure 3, M and N). However, immunoblots of whole-kidney lysates from cKO and control mice did not show an overall change in NHE3 or SGLT2 protein expression levels in cKO kidneys (Supplemental Figure 10). Although the total SGLT2 protein levels were unchanged in the *Myh9&10*-cKO kidneys, we observed a small but significant reduction in apically localized SGLT2 and excessive sodium and glucose excretion in the urine of *Myh9&10*-cKO mice (Supplemental Figure 6 and Supplemental Tables 1 and 2), indicating a decline in SGLT2 function.

### Loss of MYH9&10 proteins in mouse renal tubules results in intracellular accumulation of glycosylphosphatidylinositol-anchored protein UMOD and gradual loss of NKCC2 in the TAL.

**Figure 4:**
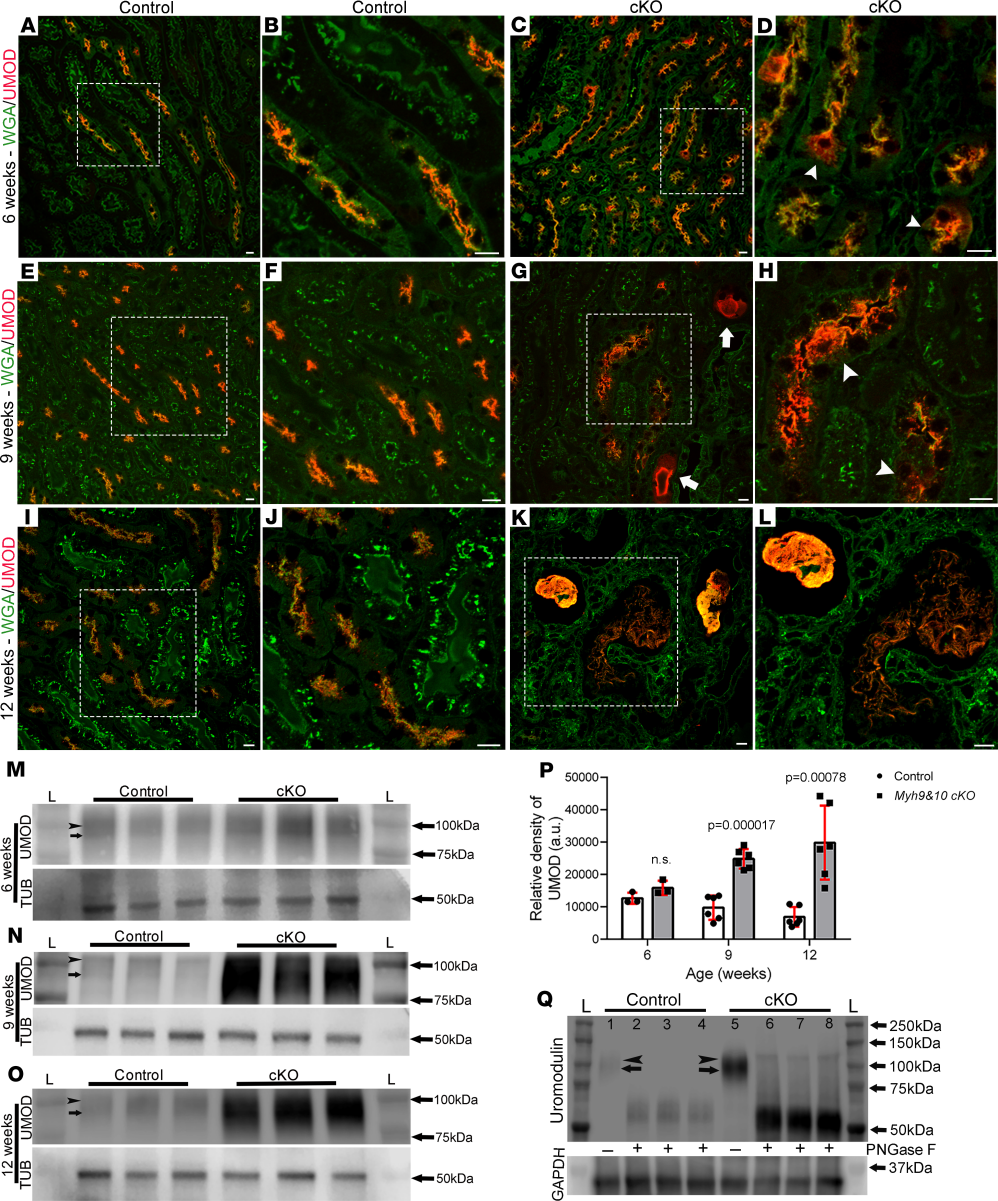
Inactivation of *Myh9&10* results in progressive mislocalization and intracellular accumulation of UMOD in the TAL. (**A**–**L**) Representative images from Bouin’s-fixed kidney sections from 6-week-, 9-week-, and 12-week-old mice stained for UMOD and WGA. Images from control kidney sections at 6 weeks (**A** and **B**), 9 weeks (**E** and **F**), and 12 weeks (**I** and **J**) show UMOD (red) localization to the apical membrane. (**C** and **D**) Six-week-old cKO kidney cells (**D**, white arrowhead) containing UMOD-positive intracellular puncta and loss of localization to the apical membrane. (**G** and **H**) Kidney sections from 9-week-old cKO mice show accumulation of UMOD in the intracellular and subapical regions (**H**, white arrowhead), as well as the lumen (**G**, white arrows). (**K** and **L**) Twelve-week-old cKO kidney sections show tubular dilation and excessive accumulation of UMOD in the luminal space. The white boxes mark the enlarged regions represented in the adjacent images. Scale bar: 10 μm. Images are representative of *n* ≥ 3 kidneys for control and cKO samples. (**M**–**O**) Whole-kidney lysate immunoblots detected both the ~100 kDa (mature, black arrowhead) and ~85–87 kDa (immature, black arrow) UMOD proteins. Tubulin (TUB) was used as loading control. (**M**) Six-week-old cKO kidney lysates show a slight increase in intensity of UMOD compared with controls. (**N** and **O**) 9-week-old cKO (**N**) and 12-week-old cKO (**O**) kidney lysates show a pronounced increase in UMOD intensity compared with controls. (**P**) Quantification of the relative density of UMOD bands observed in control and cKO kidney samples. Nine- and 12-week-old cKO samples show a statistically significant increase of UMOD compared with controls (*P* = 0.000017 and 0.00078, respectively). *n* = 6 control and 6-cKO samples at each time point. *P* values were calculated using a multiple *t* test, 2-tailed. Error bars represent standard deviation. (**Q**) Immunoblot of PNGase F–treated 12-week-old kidney lysates indicating that UMOD in both control and cKO samples is posttranslationally modified. In treated control samples (lanes 2–4, *n* = 3), diffuse bands at ~60–70 kDa are present, which correspond to the form of UMOD devoid of all N-linked oligosaccharides. In treated cKO kidney lysates (lanes 6–8, *n* = 3), PNGase F treatment also deglycosylated UMOD; however, the banding pattern is slightly lower than control samples (~50–60 kDa). One control and 1 cKO sample (lanes 1 and 5, respectively) underwent the same experimental treatment but without PNGase F enzyme and showed 2 diffuse UMOD bands (black arrowhead and arrow). “L” marks the ladder lanes, and molecular weight labels indicate the corresponding bands on the ladders.

MYH9 (NM2A) and MYH10 (NM2B) are both expressed in the mouse TAL segment (15); therefore, we assessed the localization and expression pattern of the major TAL-specific membrane-associated proteins. UMOD is a unique glycosylphosphatidylinositol-anchored (GPI-anchored) protein in the TAL segment that undergoes N-glycosylation(s) and as a mature protein has a 16- to 24-hour turnover rate on the apical membrane (22). In control kidney sections at all ages, UMOD localized to the apical membrane with WGA (Figure 4, A, B, E, F, I, and J). In the cKO kidney sections, we observed cytosolic accumulation of UMOD within some TAL tubules as early as 6 weeks of age (Figure 4, C and D, arrowheads). At 9 weeks, we observed progressive intracellular accumulation of UMOD in multiple TAL tubules (Figure 4H, arrowheads) and in the lumens of dilated tubules with thinned-out epithelial cells. (Figure 4G, arrows). In 12-week-old cKO kidney sections, tubules were severely dilated, and UMOD accumulation was observed within many of the TAL lumens (Figure 4, K and L). Immunoblots showed robust expression of UMOD as a double band (Figure 4, M–O), one at ~85–87 kDa (arrow) and another at ~100 kDa (arrowhead). These bands represent the immature (85–87 kDa) and mature (100 kDa, N-linked glycan and high mannose–modified) proteins (23, 24). In whole-kidney lysates from 6 to 12 weeks of age, we observed an age-dependent progressive increase in UMOD protein levels in the cKO compared with the controls (Figure 4, M–O). Analysis of the relative density of the bands from immunoblots confirmed a significant increase in UMOD protein levels in 9-week and 12-week-old kidneys of cKO mice (Figure 4P). Interestingly the cKO lysates showed a range of UMOD bands that were smaller than the mature ~100 kDa protein. To determine if the posttranslational modification status of the accumulated UMOD was altered in cKO kidneys, we incubated whole-kidney lysates with PNGase F, an amidase that cleaves all the N-linked glycan modifications from UMOD (24). Immunoblotting detected 2 diffuse bands of UMOD in untreated control and cKO kidney lysates (Figure 4Q). The control kidney lysates treated with PNGase F showed ~60–70 kDa diffuse bands that corresponded to the form of UMOD devoid of N-linked glycan modifications. Interestingly, the UMOD in cKO kidney lysates also showed sensitivity to PNGase F treatment, indicating the presence of N-linked glycan modifications. However, the banding pattern was slightly lower than the control samples (~50–60 kDa), indicating there may be differences in the posttranslational modifications of UMOD in cKO kidneys (Figure 4Q).

**Figure 5:**
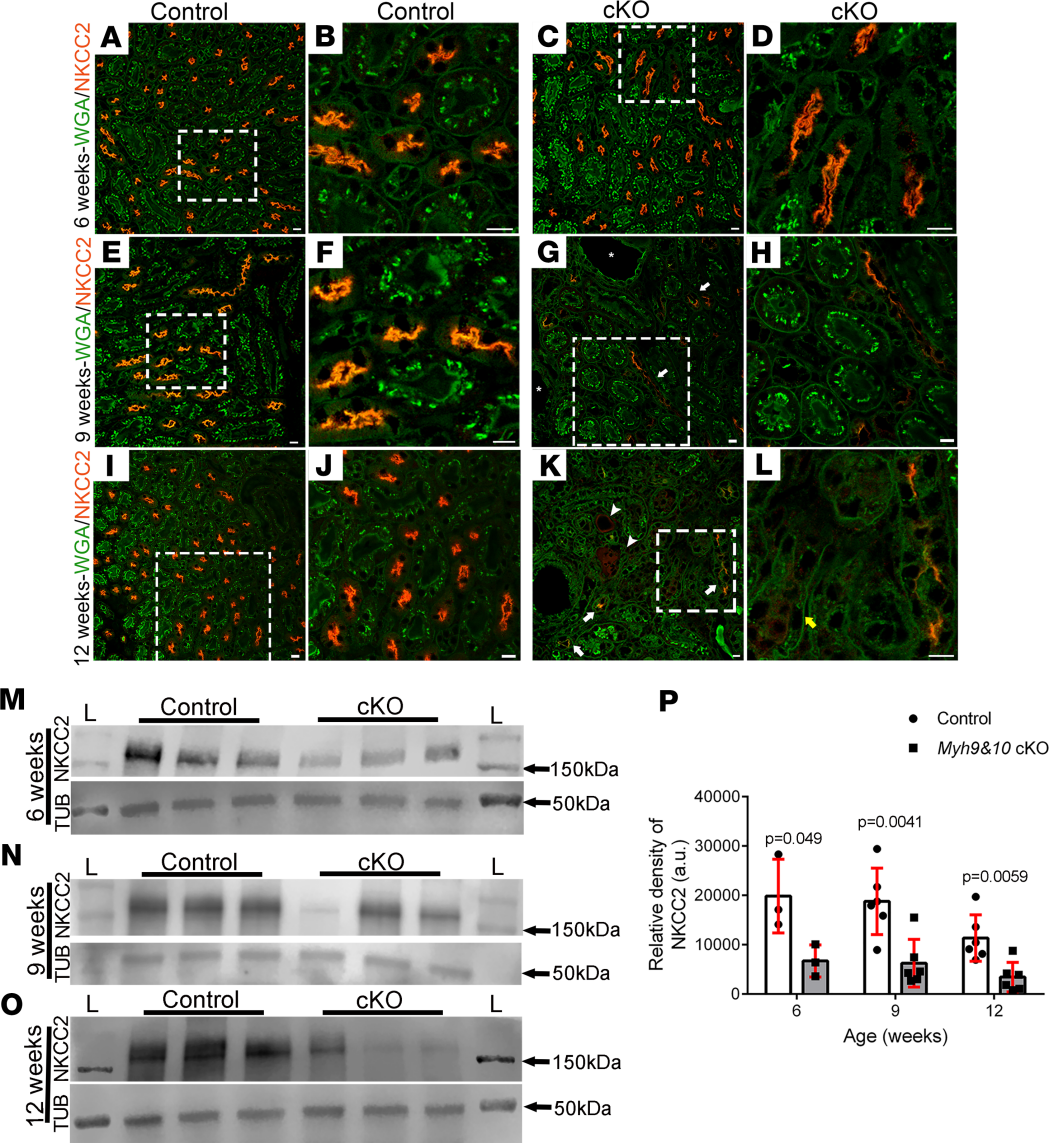
Loss of MYH9 and MYH10 in renal tubules result in gradual loss of NKCC2 from the TAL tubules. (**A**–**L**) Representative images from Bouin’s-fixed cKO mice and control littermate kidney sections from 6-week, 9-week, and 12-week cohorts were stained with NKCC2 antibody and WGA. Images from control kidney sections at 6-week (**A** and **B**), 9-week (**E** and **F**), and 12-week (**I** and **J**) time points show normal morphology of the TAL tubules with NKCC2 (red) localizing to the apical membranes. (**C** and **D**) Six-week-old cKO kidneys show TAL tubules with minimal changes in NKCC2 localization to the apical membrane. (**G** and **H**) Kidney sections from 9-week-old cKO mice show TAL tubules with partial to near-complete loss (**G**, white arrow) of NKCC2 from the apical membrane. (**K** and **L**) Twelve-week cKO kidney sections show loss of NKCC2 protein from the apical membrane of TAL tubules (**K**, white arrows); however, some tubules have accumulation of NKCC2 in the luminal space (**K**, white arrowheads) or visible intracellular NKCC2 puncta (**L**, yellow arrow). The white boxes mark the enlarged regions represented in the adjacent images. Scale bar: 10 μm. Images are representative of *n* ≥ 3 kidneys for control and cKO samples. (**M**–**O**) Whole-kidney lysates from 6-week, 9-week, and 12-week cohorts were subjected to immunoblot analysis to detect NKCC2 (~160 kDa) protein. Tubulin was used as a loading control. (**M**) The 6-week-old cKO samples show a decrease in NKCC2 protein levels compared with controls. (**N**) At 9 weeks of age, NKCC2 levels are variable in the cKO samples compared with the control kidney lysates. (**O**) The 12-week-old cKO lysates show loss of NKCC2 protein compared with control kidney lysates. “L” marks the ladder lanes; molecular weight labels indicate the corresponding bands on the ladder. (**P**) The graph shows quantification of the relative density of the NKCC2 bands observed in the control and cKO kidney samples. The 6-week-old cKO samples show statistically significant decrease in NKCC2 protein levels compared with control (*P* value = 0.049: *n* = 3 for control and cKO). The 9-week and 12-week-cKO samples show decreased relative density of NKCC2 compared with controls and were statistically significant (*P* value = 0.0041 and 0.0059, respectively; *n* = 6 for control and cKO). *P* values were calculated using multiple *t* test, 2-tailed. Error bars represent standard deviation of samples.

We next asked whether loss of NM2 isoforms leads to accumulation and/or mislocalization of other membrane-associated proteins in the TAL tubules. The localization of the basolateral sodium pump, Na^+^/K^+^-ATPase, and the apical inward rectifying potassium channel, ROMK1, were normal in kidney sections from 9-week-old control and cKO mice (Supplemental Figure 11). Interestingly, in *Myh9&10*-cKO kidneys, we observed significant changes in the expression of NKCC2, which facilitates 20%–25% of total sodium reabsorption by the kidneys (25). NKCC2 localized to the apical membrane in control kidney sections (at 6, 9, and 12 weeks) costained for the membrane marker, WGA (Figure 5, A, B, E, F, I, and J). In 6-week-old *Myh9&10*-cKO kidneys, no major changes in apical membrane localization of NKCC2 were apparent (Figure 5, C and D). However, we observed substantial loss of NKCC2 localization to the apical membrane in TAL tubules at 9 weeks and 12 weeks of age (Figure 5, G, H, K, and L, respectively). Imaging of control kidney sections stained for UMOD and NKCC2 using the ZEISS Airyscan microscope detected both proteins at the apical membrane with partial colocalization in some regions (Supplemental Video 1). In *Myh9&10*-cKO tubules that had accumulated UMOD in the tubular lumen, we observed loss of NKCC2 from the apical membrane (Supplemental Video 2). Immunoblot analysis of whole-kidney lysates confirmed the gradual loss of NKCC2 protein levels (~160 kDa) in the cKO kidneys compared with control kidneys at 6 weeks, 9 weeks, and 12 weeks of age (Figure 5, M–O, respectively). Analysis of the relative density of NKCC2 bands from immunoblots showed a statistically significant decrease in NKCC2 protein levels in the *Myh9&10*-cKO kidneys (Figure 5P). This loss of NKCC2 protein levels may explain the increased excretion of sodium and potassium in *Myh9&10*-cKO urine compared with the control. However, this salt-wasting phenomenon differed between the male and female cohorts (Supplemental Figure 6 and Supplemental Tables 1 and 2).

### ER tubule expansion is observed in Myh9&10-cKO mouse kidneys.

**Figure 6:**
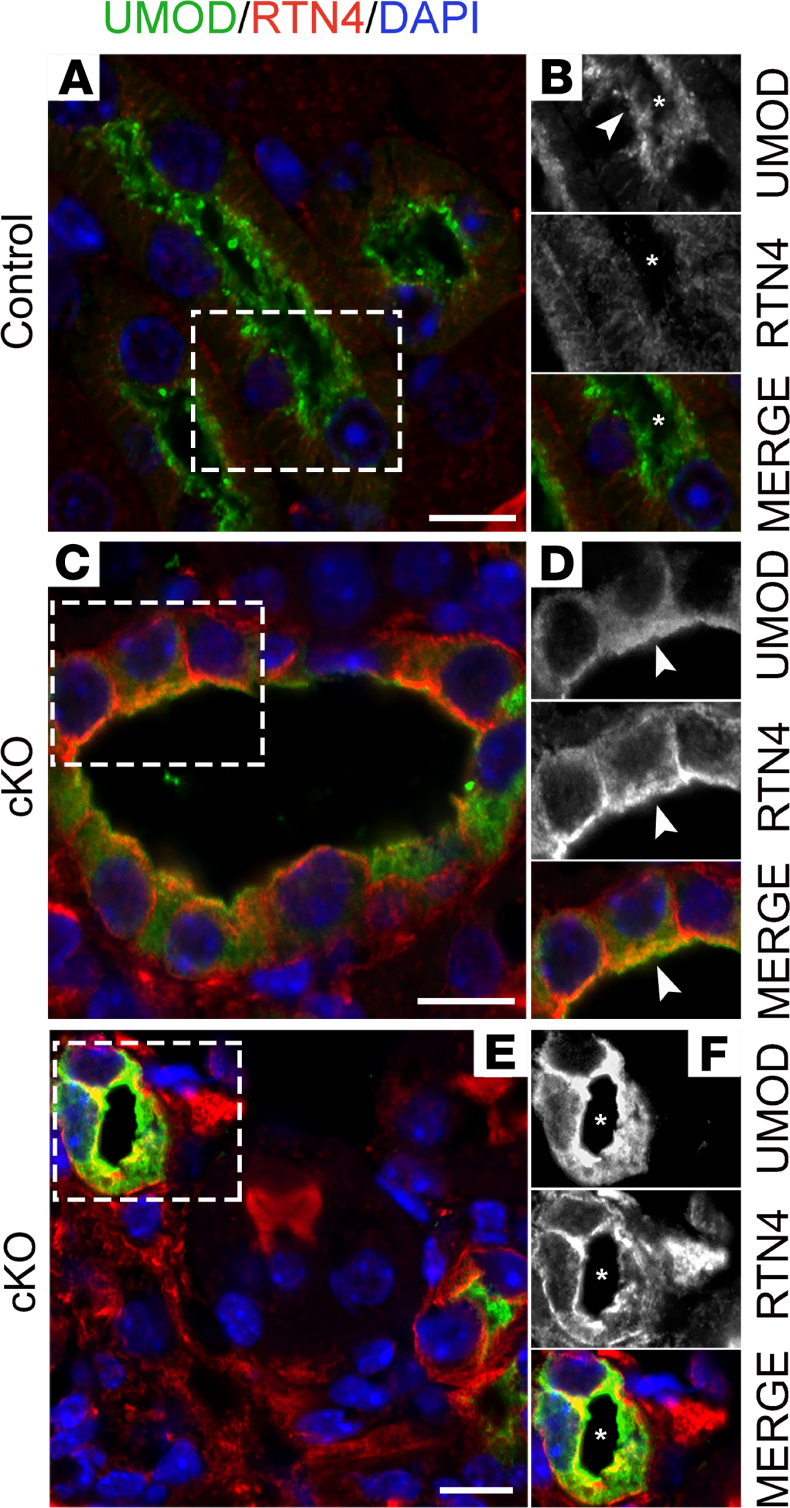
Expansion of RTN4 positive ER tubules is observed in the *Myh9&10*-cKO mouse kidneys. (**A**–**F**) Images represent paraformaldehyde-fixed (PFA-fixed) 9-week-old control and cKO kidney sections stained to visualize ER tubule–associated protein RTN4 along with UMOD and DAPI in the TAL tubules. (**A**) Control kidney section stained for RTN4 and UMOD showing discrete RTN4 staining (red) in the TAL tubules that also express UMOD (green) along the apical membrane. (**B**) Region of interest (ROI) images from a control TAL tubule in **A** (white box) indicate that UMOD localizes to the apical membrane (white arrowhead), while RTN4 appears as very thin filaments that run from the apical membrane to the basolateral membrane. (**C** and **E**) cKO kidney sections show increased intensity of RTN4 staining in the cKO TAL cells that partially colocalized with UMOD in several regions of the cell. UMOD accumulation within the cells is apparent. (**D** and **F**) ROI images of cKO TAL tubules represented in **C** and **E** (white box) show changes in RTN4 and UMOD expression and localization. Gray scale and merged images show the increase in RTN4 and UMOD intracellular staining in the cKO TAL tubule, as well as regions of colocalization along the membrane and inside the cell. Asterisks (*) in all images denote the lumen, and white arrowhead marks the apical membrane. Scale bar: 10 μm. Images are representative of analysis of sections from 3 control and cKO kidneys (*n* = 3).

**Figure 7:**
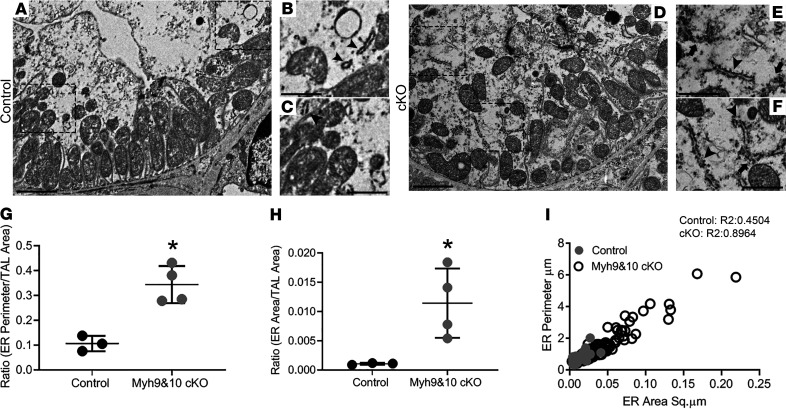
TEM confirms ER expansion in *Myh9&10*-cKO mouse TAL tubules. (**A**–**F**) Nine-week-old control and cKO mouse kidneys were subjected to TEM to analyze the ultrastructure of the TAL tubules. (**A**–**C**) Representative TEM image from the control mouse kidney showing the ultrastructure of the TAL epithelial cells. Black boxes represent the selective regions of the TAL epithelium in **A** for visualization of ER tubules shown in **B** and **C** (arrowheads). The representative ER tubules in the control kidney have ribosomes, are short in length, and have a small ER lumen (black arrowheads). (**D**–**F**) Representative TEM images showing a TAL tubule from the cKO mouse kidney. Black boxes represent the selective regions of the TAL epithelium in **D** for visualization of ER tubules shown in **E** and **F**. ER tubules have increased length and lumen diameter (luminal space) in the cKO TAL tubules (black arrowheads). Some ER tubules appear to be disintegrating and no longer maintain normal structure (black arrows). Scale bars: 2 μm (**A** and **D**), 1 μm (**B**, **C**, **E**, and **F**). (**G**–**I**) Graphs show the relationship between ER perimeter, ER area, and total TAL tubule area from 3 tubules (17 TAL cells, 181 ER tubules) from the control kidney and 4 tubules (28 TAL cells, 358 ER tubules) from the cKO kidney. (**G**) cKO kidneys have a statistically significant (*) increase in the ratio of ER perimeter to the total area of the TAL tubules compared with the controls (*P* value = 0.003). (**H**) The ratio of total ER area to TAL tubule area varied between the cKO tubules, ranging from 5- to 20-fold increase in the ratio compared with the control tubules and was significantly (*) different (*P* value = 0.031). (**I**) Scatter plot showing the relationship between ER perimeter and ER area in the TAL tubules of control and cKO kidneys with a linear relationship between perimeter size and ER area. Statistics were done using unpaired *t* test, 2-tailed. Error bars depict standard deviation.

Mutations in the UMOD gene in humans result in autosomal dominant tubular kidney disease (ADTKD) because of accumulation of the mutant protein within the ER, which results in activation of ER stress–mediated injury (26, 27). In order to identify whether the excessive UMOD in *Myh9&10*-cKO resulted in changes to ER, we costained kidney sections using an ER tubule–associated structural protein, reticulon-4 (RTN4), and UMOD antibodies. In control kidneys, UMOD localized predominately to the apical membrane, while RTN4 protein localized as very thin intracellular filaments in the cytosol (Figure 6, A and B). In cKO kidney sections, we observed increased RTN4-positive ER tubules along the membranes and in the cytosol that colocalized with UMOD (Figure 6, C–F). Imaging using the ZEISS Airyscan microscope further confirmed increased expression and intracellular accumulation of UMOD- and RTN4-positive ER tubules in *Myh9&10*-cKO kidneys (Supplemental Video 4) compared with the control (Supplemental Video 3). We also used transmission electron microscopy (TEM) to assess changes in ER structure in *Myh9&10*-cKO and control kidneys. TEM analysis of TAL tubules and cells from 9-week-old cKO kidneys showed cells filled with expanded ER tubules that had increased length and diameter (Figure 7, D–F) compared with control sections (Figure 7, A–C). Some ER tubules no longer maintained normal structure and appeared to be disintegrating (Figure 7, E and F, arrow). Quantitative analysis of ER structures within multiple TAL tubules showed significant increase in both area and perimeter of ER tubules in the cKO TAL cells (Figure 7, G–I).

### ER stress and unfolded protein responses are activated in Myh9&10-cKO kidneys.

**Figure 8:**
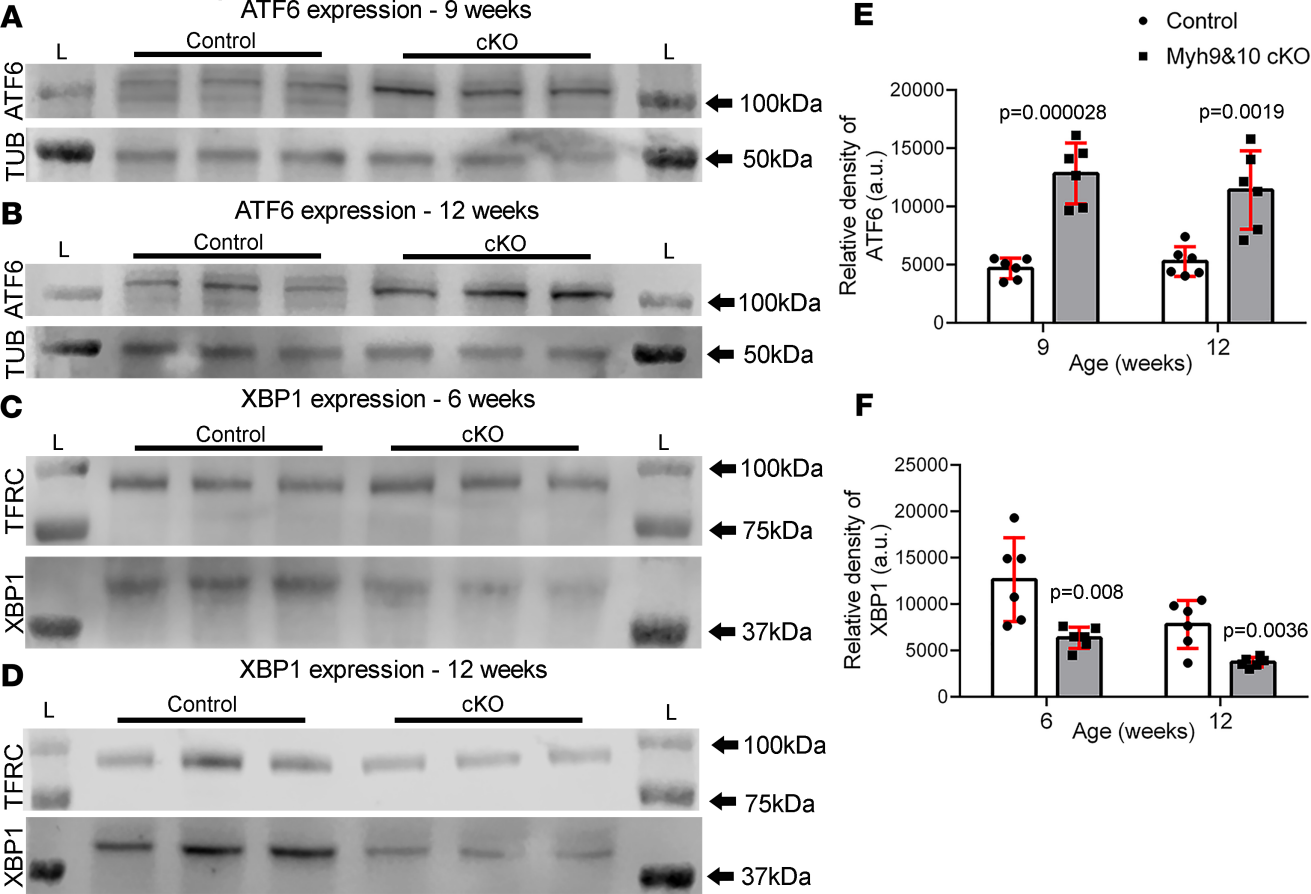
ER stress and UPR pathway is activated in *Myh9&10*-cKO kidneys. (**A** and **B**) Whole-kidney lysates from 9-week and 12-week cohorts were subjected to immunoblot analysis to detect protein levels of the ER stress protein ATF6 and tubulin (loading control). Immunoblot analysis detected a ~109 kDa band for ATF6 in control and cKO kidneys. Intensity of the ATF6 bands was increased in both 9-week- and 12-week-old cKO samples. (**E**) The graph shows relative density of ATF6 bands indicating a statistically significant increase in ATF6 expression in cKO kidneys at both the 9-week and 12-week time points (*n* = 6, *P* value = 0.000028 and 0.0019 respectively, using a multiple *t* test, 2-tailed). Error bars depict standard deviation. (**C** and **D**) Whole-kidney lysates from 6-week and 12-week cohorts were subjected to immunoblot analysis to detect protein levels of the UPR pathway protein XBP1 and TFRC (loading control). Immunoblot analysis detected a ~40 kDa band for XBP1 in control and cKO kidneys. Intensity of the XBP1 bands is decreased in both 6-and 12-week-old cKO samples compared with the controls. (**F**) The graph shows relative density measurements indicating a decrease in XBP1 protein expression in cKO kidneys, which is progressive and statistically significant at 6-week and 12-week time points (*n* = 6, *P* value = 0.008 and 0.003, respectively, using multiple *t* test, 2-tailed). “L” marks the ladder lanes, and molecular weight labels indicate the corresponding bands. Error bars depict standard deviation.

To evaluate whether ER stress and unfolded protein response (UPR) are activated, we assessed both the activating transcription factor 6 (ATF6) and inositol-requiring transmembrane kinase/endoribonuclease 1α/X-box binding protein 1 (IRE1α/XBP1) ER stress pathway components in control and *Myh9&10*-cKO kidneys. Immunoblot analysis of control and cKO kidney lysates showed a statistically significant increase in levels of the ER stress protein ATF6 in the cKO kidneys, at 9 and 12 weeks of age (Figure 8, A, B, and E), indicating activation of the UPR pathway and ER stress. The IRE1α/XBP1 pathway is active during adaptive phase and attenuated during apoptotic phase of ER stress response (28). The immunoblot analysis showed progressive decline in XBP1 protein levels in cKO kidneys from 6 to 12 weeks of age compared with the control littermates (Figure 8, C, D, and F), supporting the activation of the ER stress response in *Myh9&10*-cKO mouse kidneys.

### Localization and expression of ER chaperone proteins are altered in Myh9&10-cKO kidneys.

**Figure 9:**
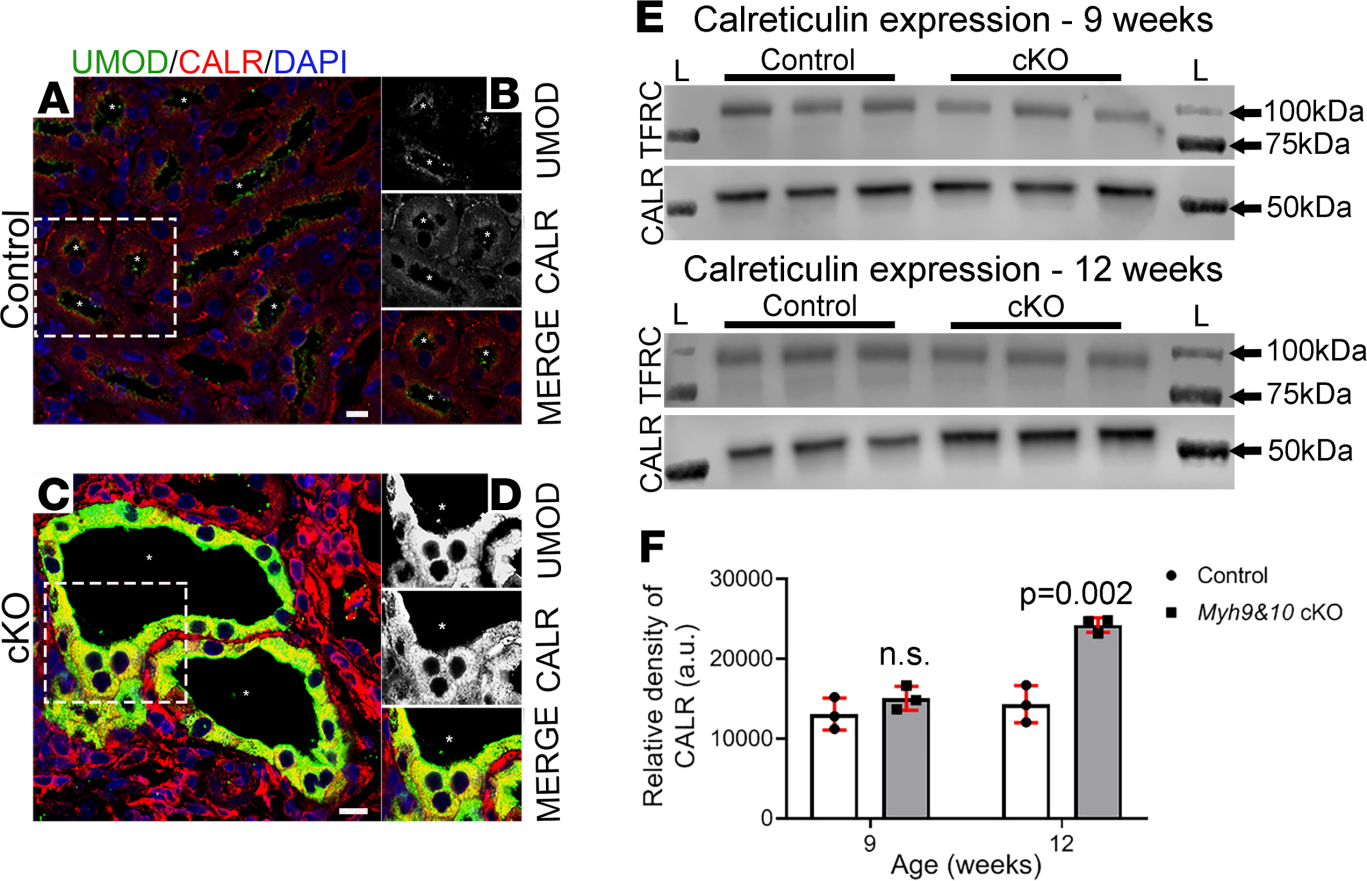
Expression of ER chaperone calreticulin is increased in *Myh9&10*-cKO mouse kidneys and partially colocalizes with UMOD-positive vesicles. (**A**–**D**) Images represent PFA-fixed, 9-week-old control and cKO kidney sections stained to visualize ER chaperone protein calreticulin (CALR) along with UMOD and DAPI in the TAL tubules. (**A**) Cross section of tubules from control kidney shows expression and localization pattern of CALR (red) along with UMOD (green). (**B**) Enlarged region from **A** (white box) reveals that in control kidneys, UMOD localizes to the apical membrane of the TAL tubule, and CALR localizes to the nuclear membrane as well as intracellular punctate structures. (**C**) Dilated tubules from the cKO kidney sections show excessive UMOD accumulated within the cells; colocalization of vesicular structures positive for CALR along with UMOD is apparent in some areas inside the cell. (**D**) Enlarged region from C (white box) depicts the partial colocalization between CALR and UMOD-positive vesicles around the nucleus in the TAL cells. Asterisks (*) in all images denote the lumen, which is dilated in the cKO kidneys. Scale bar: 10 μm. Images are representative of *n* ≥ 3 kidneys for control and cKO samples. (**E**) Whole-kidney lysates from cKO and control mice from 9-week and 12-week cohorts were subjected to immunoblot analysis to detect protein levels of CALR and TFRC (loading control). Immunoblot analysis detected a ~50 kDa band for CALR in control and cKO kidneys. Intensity of the CALR bands was increased in the 12-week-old cKO samples, but not in 9-week samples. “L” marks the ladder lanes, molecular weight labels indicate the corresponding bands. (**F**) The bar graph shows that calreticulin expression is increased in cKO kidneys and is statistically significant at the 12-week time point (*P* value = 0.002 by multiple *t* test, 2-tailed; *n* = 3 samples per group at each time point). Error bars represent standard deviation.

Deregulation of biosynthetic pathways and activation of ER stress can result in changes to ER quality control proteins that handle the overload of misfolded proteins. In mammalian cells, GPI-anchored and N-glycosylated protein quality control is regulated by the ER chaperone proteins calnexin and calreticulin (29, 30). Typically, GPI-anchored proteins prefer to associate with the membrane-bound calnexin; however, interactions with the soluble calreticulin are also observed (31). Because of the accumulation of UMOD, which is both a GPI-anchored and N-glycosylated protein, we analyzed the expression and association of calnexin and calreticulin in *Myh9&10*-cKO and control mouse kidneys using immunostaining and immunoblotting methods. In 9-week-old control kidney sections, calreticulin localized to small punctate structures within the cells, while UMOD predominantly localized to the apical membrane (Figure 9, A and B). In cKO kidneys, dilated TAL tubules had increased expression of calreticulin-positive vesicular structures, some of which colocalized with the excess UMOD (Figure 9, C and D). Imaging using the ZEISS Airyscan microscope supported colocalization between calreticulin and UMOD in *Myh9&10*-cKO kidneys (Supplemental Videos 5 and 6). Immunoblot analysis of whole-kidney lysates showed a statistically significant increase in calreticulin protein levels at 12 weeks of age (Figure 9, E and F).

**Figure 10:**
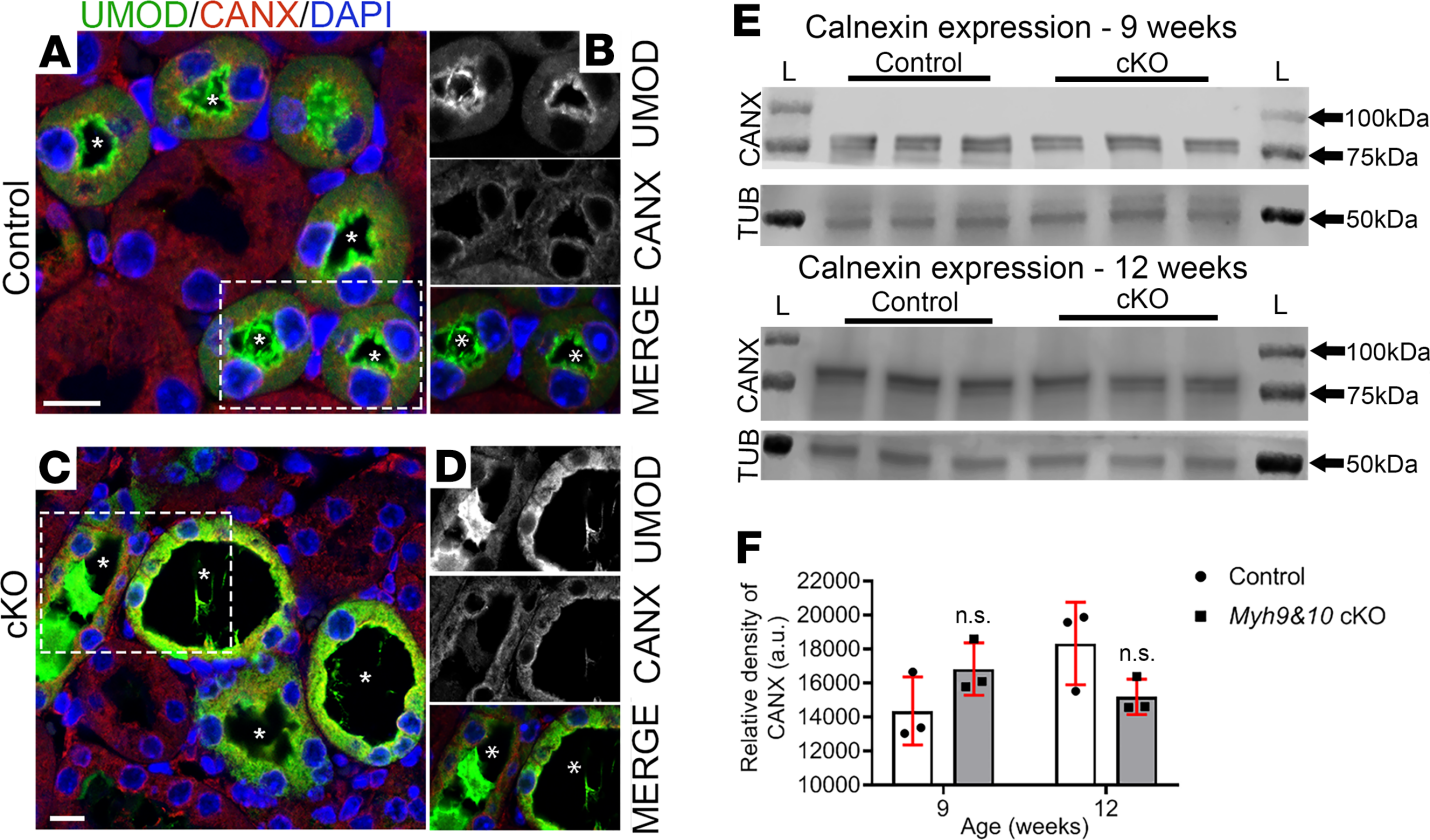
ER chaperone protein calnexin localization is altered in *Myh9&10*-cKO kidneys. (**A**–**D**) Images represent 9-week-old, PFA-fixed, control, and cKO kidney sections stained to visualize ER chaperone protein calnexin (CANX) along with UMOD and DAPI in the TAL tubules. (**A**) Tubules from control kidney show expression and localization pattern of CANX (red) along with UMOD (green). (**B**) Enlarged regions from **A** (white box) show that UMOD localizes to the apical membrane of the TAL tubule, and CANX localizes to the nuclear membrane and as intracellular punctate structures. (**C**) Dilated tubules from the cKO kidney sections show UMOD accumulation, and CANX localizes within the cytosol instead of along the nuclear membrane in TAL cells. (**D**) Enlarged region from **C** (white box) and individual channel insets depict the altered localization of CANX and accumulation of UMOD in the TAL cells; no colocalization is detected. Asterisks (*) in all images denote the lumen. Scale bar: 10 μm. Images are representative of *n* ≥ 3 kidneys for control and cKO samples. (**E**) Whole-kidney lysates from cKO and control mice from 9-week and 12-week cohorts were subjected to immunoblot analysis to detect protein levels of calnexin (~75 kDa) and tubulin. No significant changes in calnexin expression levels were observed. “L” marks the ladder lanes; molecular weight labels indicate the corresponding bands. (**F**) The bar graph shows quantification of the relative density of the CANX bands detected from 9-week- and 12-week-old cohorts and does not show any significant changes between control and cKO sample (*P* value: 0.16 and 0.10, respectively; *n* = 3 samples each). *P* values were calculated using multiple *t* test, 2-tailed. Error bars depict standard deviation.

Calnexin localized along the nuclear membrane and in intracellular punctate structures in the 9-week-old control kidneys (Figure 10, A and B). In *Myh9&10*-cKO kidneys we observed intracellular and subapical localization of calnexin, which did not colocalize with the excess UMOD (Figure 10, C and D). Immunoblot analysis of 9-week and 12-week-old whole-kidney lysates from control and cKO mice did not show an increase in calnexin protein expression (Figure 10, E and F). Taken together these results suggest alterations in ER chaperone localization and expression in *Myh9&10*-cKO kidneys.

## Discussion

NM2 belongs to the class II family of conventional myosins that self-organize into nonsarcomeric, force-generating filaments of varying sizes, number, and polarity, making it a versatile system for regional and cell type–specific control of molecular pathways (32–34). Based on our previous identification of novel roles for MYH9&10 proteins in membrane remodeling during clathrin-mediated endocytosis (6, 7), we hypothesized that NM2 proteins might play a critical role in renal epithelial transport pathways. Consistent with this idea, we identified a distinct expression and membrane localization pattern for NM2 proteins in murine renal tubules (15). While our primary motivation was to test the role for *Myh9&10* genes in renal epithelia and to use kidney as a model organ to study NM2-mediated specialized cellular transport pathways, we were also influenced by the fact that mutations in the *MYH9* gene are associated with end-stage renal disease in human patients (35–37). Approximately one-third of patients with *MYH9*-related disorder have kidney dysfunction (35–37) characterized by progressive proteinuria, glomerulosclerosis with foot process effacement, and kidney failure (36–38). Therefore, prior *MYH9* studies in the kidney concentrated on podocyte biology and the glomerular filtration barrier to elucidate the underlying mechanism of disease in these patients (39, 40). Genetic inactivation of *Myh9* in podocytes of mice indicated strain-dependent variations in which mild podocyte injury and foot process effacement were observed; however, none of the models developed severe CKD (41, 42). Recently, increased proteinuria, podocyte injury, and focal segmental glomerulosclerosis have been observed in a mouse model harboring MYH9 E1841K mutation in response to a high-salt diet (43). Furthermore, localization pattern of MYH9 in podocytes using electron microscopy and other super resolution microscopy methods showed MYH9 expression is limited to the cell body of podocytes and not in the foot process and that podocyte injury causes redistribution of MYH9 protein to the foot processes (44). This report also proposed a potentially novel model of concerted action between MYH9-positive contractile filaments in the cell body and the MYH9-negative noncontractile filaments in the foot process for maintenance of podocyte structure and function (44). Taken together, it is possible that while *MYH9* plays a role in podocyte function, depletion of MYH9 in podocytes may not have a serious effect on kidney function unless combined with an additional hit/stress to the podocytes, and this second hit could in theory originate from tubular injury and tubulointerstitial disease.

We hypothesized that in *MYH9*-associated kidney disease the underlying defect might be a deregulated epithelial transport pathway that triggers tubular injury–associated disease, which in turn can lead to glomerular defects in the latter stages. To test this, we generated mice homozygous for *Myh9*- and *Myh10*-floxed alleles that also harbored the *Pax8*→*rtTA* driver and a tetracycline-responsive Cre (*Tet-O-Cre*). This strategy allowed for spatial and temporal control of gene knockout selectively in renal epithelial cells, which was important because of critical roles for MYH9&10 in brain and heart development, platelet biogenesis, and myriad other cell type–specific functions (45–51). Moreover, in *MYH9-*related disorder in patients with kidney disease, autosomal dominant mutations are located in the motor domain or the coiled-coil rod domain of the protein and result in loss of motor activity or disruption of filament formation, producing a dominant negative effect by interfering with WT NM2 protein function (52). We decided to inactivate both *Myh9* and *Myh10* to model the most severe loss of NM2 renal epithelial phenotype by uncovering the redundant roles for MYH9 and MYH10 in renal epithelial segments. Our results demonstrate that the loss of MYH9 and MYH10 in adult mouse renal epithelium results in progressive kidney disease of tubular origin, as evidenced by an increase in tubular injury markers, NGAL and KIM1, along with dilation of tubules, increased immune cell infiltration, and increased BUN and serum creatinine. *Myh9* inactivation by itself resulted in a later onset progressive kidney disease in mice but with decreased severity and mortality (data not shown; unpublished observations).

One of the earliest defects we observed following *Myh9&10* inactivation was the accumulation of UMOD inside the TAL epithelium, which led us to compare the observed phenotype with the previously described ADTKD caused by mutations in UMOD (ADTKD-UMOD) (26, 27, 53). Human, transgenic mouse, and cell culture–based studies show that the underlying mechanism of ADTKD-UMOD is ER retention of mutant protein that causes ER stress and tubular injury (27, 54–56). In *Myh9&10*-cKO mouse kidneys, we observed expansion of RTN4-positive ER tubules, along with activation of ER stress and UPR pathways. Interestingly, our experiments also demonstrated a gradual loss of NKCC2 in the TAL segment, along with increased urinary sodium and potassium in *Myh9&10*-cKO mice. We observed colocalization of NKCC2 with calnexin in *Myh9&10*-cKO kidneys (Supplemental Figure 12), and this association might target NKCC2 to the ER-associated protein degradation (ERAD) pathway, leading to loss of NKCC2 seen in *Myh9&10*-cKO mice (57). Previous reports have shown that loss of UMOD has direct effects on the concentration of NKCC2 and ROMK1 on the apical membrane (56, 58, 59). It is possible that MYH9&10 are directly involved in regulating apical membrane localization and endocytosis and exocytosis of NKCC2, or indirectly due to UMOD accumulation and deregulated biosynthetic pathway. Further studies are necessary to identify the molecular link between MYH9&10, UMOD, and NKCC2 proteins.

We observed minor changes in the expression of sodium transporters in the proximal tubular segments; both NHE3 and SGLT2 staining were reduced in individual tubules in *Myh9&10*-cKO kidneys. It is possible that the proximal tubule phenotype is mitigated by the expression of a third NM2 gene, *Myh14*, that may compensate for the loss of *Myh10*. Similarly, in the murine distal convoluted tubules (DCTs) and collecting duct segments of the renal tubules, MYH9 is not expressed and MYH10 is predominantly expressed and localized to cell-cell adhesions and tight junctions (15), where it might perform more traditional roles of NM2 proteins. Analysis of DCT-specific phosphorylated sodium chloride cotransporter and connecting segment/collecting duct–associated water channel aquaporin-2 did not demonstrate any changes in localization pattern but subtle changes in expression levels (Supplemental Figure 13). TAL-, PCT-, and distal nephron–specific conditional inactivation of NM2 proteins in mice will further address the tubular segment–specific transport defects in the kidney.

We attribute the TAL-specific cellular phenotype in *Myh9&10*-cKO mice to the loss of both NM2 proteins expressed in the TAL segment, leaving it unable to rescue the UMOD transport defect. Our data suggest that UMOD delivery to the plasma membrane is dependent on NM2 activity. We speculate that NM2 proteins play unique role(s) along the plasma membrane as well as ER transport pathway to coordinate anterograde transport of UMOD. Whether this NM2-mediated transport is unique to UMOD, or is a common pathway for all GPI-anchored proteins in specific cell types, needs to be tested in future experiments. However, the severity of UMOD accumulation within the TAL epithelium may be attributed to (a) the 24-hour turnover rate that demands continuous biosynthesis and delivery to the membrane (22), (b) differences in protein quality control pathways such as ERAD (60) versus rapid ER-stress induced export (30) in efficiently handling the accumulated cargo, and (c) sustained activation of hormonal or salt-induced signals that regulate the biosynthesis of UMOD. Future work will need to address molecular mechanisms by which NM2 proteins regulate GPI-anchored protein (e.g., UMOD) versus transmembrane protein (e.g., NKCC2) transport, including the identification of the subcellular site(s) of action along the vesicular transport pathway and NM2 interacting protein partners.

In order to relate our findings to human renal epithelium, we assessed the expression pattern of NM2 proteins in the renal tubules of the human donor kidneys using immunostaining methods (Supplemental Figure 14). Our results show distinct as well as overlapping cell type–specific expression and localization of MYH9 and MYH10 proteins in apical and basolateral membranes of the renal tubular segments of the human kidney (Supplemental Figure 14); MYH14 is not expressed in the tubular segments of human kidneys (Supplemental Figure 14). This raises the possibility of copolymerization of MYH9 and MYH10 proteins (61, 62) and perhaps motor dead MYO18A (63) in human renal epithelial cells that might dictate the novel and unconventional functions of these myosins in human and other mammalian kidneys. In conclusion, our work demonstrates that loss of NM2 proteins in adult mouse renal epithelium results in an overloaded biosynthetic pathway followed by ER stress and UPR activation that causes tubular injury and progressive kidney disease.

## Methods

### Mice.

Mice were generated for a renal tubule–specific, inducible cKO of *Myh9* and *Myh10* using the transgenes *Pax8*→*rtTA* and *Tet-O-Cre* (16). Control mice were littermates that carried either *Pax8*→*rtTA* or *Tet-O-Cre*, as well as mice that carried neither transgene. Mice containing the conditional alleles for *Myh9^fl^* and *Myh10^fl^* were obtained from Robert Adelstein, National Heart, Lung, and Blood Institute, NIH, Bethesda, Maryland, USA (50, 64). *Myh9*- and *Myh10*-cKO characterization was on a mixed C57BL/6 and 129 genetic background. Both female and male mice were analyzed; if not explicitly stated, both female and male data are included in the analysis. All data presented are from mice between the ages of 4 and 12 weeks of age; exact ages of data collection are listed in the figures and tables.

Doxycycline (1 mg/mL, MilliporeSigma) was administrated within drinking water supplemented with 5% sucrose from 4 to 8 weeks of age. Urine was collected using metabolic cages for 24 hours supplemented with 1% sucrose water at 4, 6, 9, and 12 weeks of age.

### Serum and tissue collection.

Serum and kidneys were collected from mice at 6, 9, and 12 weeks of age. Mice were anesthetized with isoflurane before the cardiac puncture for blood collection. Kidneys were harvested postexsanguination and were either flash-frozen in liquid nitrogen or fixed in Bouin’s fixative or 4% PFA.

### Urine and serum analysis.

Urine osmolality was determined using a VAPRO Vapor Pressure Osmometer (WESCOR). The ADVIA 1800 chemistry system (Siemens) was used to analyze serum samples for glucose (GLUH_3 kit), albumin (ALB, B01412101), creatinine (03039070), and BUN (03040257), as well as urinary total protein (05000171), urine glucose hexokinase (05001429), and urinary sodium (electrode) and potassium (electrode) concentrations.

### Determination of NGAL levels.

NGAL was measured using the Mouse Lipocalin-2/NGAL ELISA kit (MLCN20) from R&D Systems, Bio-Techne. The assay was performed following the protocol and using reagents provided with the kit. Serum samples were diluted 100-fold as suggested. Urine samples were diluted 40-fold. Standards were plotted linearly by plotting log of the lipocalin-2 concentrations and log of the optical density. Three female and 3 male control samples and 3 female and 3 male cKO samples were analyzed for each of the time points.

### Histology, immunohistochemistry, and image analysis.

Tissues fixed in PFA were stained for H&E while tissues fixed in Bouin’s fixative were stained for PAS and Masson’s trichrome. Immunofluorescence analysis was performed following the protocol from Otterpohl et al. (15). Tissues fixed in Bouin’s were used to visualize membrane-associated proteins and structures; the central lumen is closed in Bouin’s-fixed kidneys with overlapping apical membranes in the cross section. PFA-fixed tissues were used to visualize intracellular and ER proteins, and the central lumen is open and distinct. A list of primary antibodies used in this study can be found in Supplemental Table 4. Secondary antibodies conjugated to CY3, Alexa Fluor 488, CY5, DyLight 549, or Alexa Fluor 647 were procured from Jackson ImmunoResearch; Life Technologies, Thermo Fisher Scientific; or Vector Laboratories. A list of secondary antibodies can be found in Supplemental Table 5. Coverslips were mounted using fluorescence mounting medium with DAPI (Vector Laboratories), homemade Mowiol mounting medium, or ProLong Gold (Thermo Fisher Scientific).

Sections were imaged using Nikon A1 confocal microscopes. Immunofluorescence images were quantified using ImageJ software (NIH). ROIs were selected, marking the tubular area, and measurements of each ROI included area, mean intensity, and integrated density. Several ROIs per image were used to correct for background fluorescence. Total fluorescence for each tubule was calculated as *integrated density – (area* × *average background mean intensity)*. Airyscan imaging was performed in super resolution mode on a ZEISS LSM 880 Airyscan microscope equipped with a ×63 1.4 NA objective. Raw data were processed using Airyscan processing in “auto strength” mode with Zen Black software version 2.3.

### Quantification of CD3^+^ T cells and F4/80^+^ macrophages.

PFA-fixed kidney sections were stained for WGA and CD3 or F4/80 following the above protocol. Sections were imaged on the Nikon A1 using the ×40 objective (images are 318.2 × 318.2 μm). At minimum, 10 images were taken from the cortical region and 10 from the medullary region of each stained kidney section. In total 4 control kidneys (81 fields) and 4 cKO kidneys (82 fields) were analyzed for CD3^+^ T cell counts and 3 control (64 fields) and 3 cKO (60 fields) analyzed for F4/80^+^ macrophages. Counts were performed manually using ImageJ.

### Protein lysate preparation and immunoblotting methods.

Kidney lysates were made from flash-frozen whole kidneys according to previously published protocols (56). Lysates were quantified using the BCA assay kit (Pierce, Thermo Fisher Scientific), and 20 μg was loaded on 10% precast gels (Bio-Rad). Membranes were cut into strips and probed for the protein of interest and loading control with antibodies listed in Supplemental Table 4. PNGase F (P0704, New England Biolabs) treatment of whole-kidney lysates (20 μg) was performed according to manufacturer protocol, and lysates plus PNGase F were incubated at 37°C for 2 hours. For relative density analysis, immunoblots were first analyzed for any areas of overexposure using the LI-COR Odyssey software. Exported.tiff images were then converted to 32 bit in ImageJ, and the Gel analysis tool in ImageJ was used to determine the relative density of the bands. Uncut immunoblots can be found in supplemental material.

### KIM1 detection in urine samples.

The presence of KIM1 in urine of 9-week-old cohorts was determined by loading equal volumes of urine and 2× Laemmli buffer (30 μL loaded) into 10% precast acrylamide gels (Bio-Rad). Three control and 3 cKO male urine samples were analyzed.

### Transmission electron microscopy.

The kidneys were perfusion fixed in 2% PFA and 2.5% glutaraldehyde in 100 mM cacodylate buffer, pH 7.4. The kidney tissues were postfixed and processed using standard procedures. Sections of 70 nm thickness were cut with an ultramicrotome (RMC Powertome XL), and images were taken on a JEOL JEM-2100 LaB_6_ transmission electron microscope. The ER area, ER perimeter, and total TAL tubular area were measured precisely with the help of a freehand drawing tool in ImageJ (Fiji).

### Statistics.

SAS, a software suite developed for multivariate analysis, was used to perform 2-tailed unpaired *t* tests for the urine and serum analyses. For MFI analyses and Western blot quantification, we performed standard or multiple *t* test using GraphPad software. The resulting *P* values are indicated in the text and tables. *P* ≤ 0.05 was considered significant.

### Study approval.

Sanford Institutional Review Board approved our study protocol of using deidentified human kidney samples of deceased organ donors received from the South Dakota Lions Eye and Tissue Bank as Not Human Research. All experiments involving mice were approved by the Sanford Research Institutional Animal Care and Use Committee.

## Author contributions

KLO performed most of the experiments presented in the manuscript, prepared figures, and assisted in writing the manuscript. BWB performed immunoblots and other biochemical analysis. IR performed TEM experiments, supervised by PA. RGH provided technical support for the work. KRH performed immunostaining of human kidneys. CME performed tissue paraffin embedding and sectioning for all the kidney tissues. CLP was the consultant nephropathologist and along with BAM analyzed the histopathology slides. BAM also provided advisory help. JRB assisted with super resolution imaging of stained kidney sections and performed image analysis. KS assisted with design of the experimental strategy and manuscript preparation. IC designed the study, performed some microscopy experiments, analyzed results, and wrote the manuscript.

## Supplementary Material

supplemental data

supplemental video 1

supplemental video 2

supplemental video 3

supplemental video 4

supplemental video 5

## Version 1 10/01/2020

In-Press Preview

## Version 2 11/05/2020

Electronic publication
