# Supplementary material for: Conditional Myh9 and Myh10 inactivation in adult mouse renal epithelium results in progressive kidney disease
Source: JCI Insight. 2020 Nov 5;5(21):e138530. doi: 10.1172/jci.insight.138530 (PMC7710296; doi:10.1172/jci.insight.138530)

SUPPLEMENTAL TABLE 1. Metabolic measurements for female control and Myh9&10 conditional knockout mice.

	4 Weeks (Pre-Doxycycline)			6 weeks			9 weeks			12 weeks		
	Control n = 12	Myh9; Myh10 cKO n = 8	Sig	Control n = 14	Myh9; Myh10 cKO n = 10	Sig	Control n = 7	Myh9; Myh10 cKO n = 9	Sig	Control n = 5	Myh9; Myh10 cKO n = 5	Sig
**Weight g ± SD**	12.53 ± 1.80	11.01 ± 2.12	N.S	16.69 ± 1.27	15.80 ± 1.22	N.S	18.32 ± 2.11	17.15 ± 1.69	N.S	20.89 ± 2.42	16.98 ± 1.53	0.016
**Water drank ml/g ± SD**	0.47 ± 0.17	0.60 ± 0.17	N.S	0.41 ± 0.12	0.39 ± 0.16	N.S	0.34 ± 0.07	0.42 ± 0.10	N.S	0.37 ± 0.15	0.40 ± 0.08	N.S
**Urine ml/g ± SD**	0.054 ± 0.047	0.036 ± 0.052	N.S	0.011 ± 0.019	0.033 ± 0.042	N.S	0.036 ± 0.029	0.059 ± 0.071	N.S	0.050 ± 0.042	0.064 ± 0.036	N.S
**PH ± SD**	6.36 ± 0.39	6.08 ± 0.20	N.S	6.25 ±0.27	6.00 ± 0.41	N.S	6.25 ± 4.18	5.28 ± 0.26	0.001	6.30 ± 0.27	5.00 ± 0.00	<0.001
**Osmolality mmol/kg ± SD**	1547.18 ± 1158.56	2259.17 ± 1601.97	N.S	1594.40 ± 606.23	1520.14 ± 1206.69	N.S	910.20 ± 177.12	2512.67 ± 1872.38	0.033	1736.20 ± 1806.97	981.00 ± 375.82	N.S
**Total urinary protein mmol/min ± SD**	0.0007 ± 0.0004	0.0007 ± 0.0006	N.S	0.0012 ± 0.0007	0.0016 ± 0.0016	N.S	0.0009 ± 0.0004	0.0068 ± 0.0040	0.002	0.0012 ± 0.0009	0.0019 ± 0.0014	N.S
**Sodium urinary levels mmol/min ± SD**	0.046 ± 0.016	0.039 ± 0.015	0.042	0.041 ± 0.025	0.042 ±0.009	N.S	0.039 ± 0.018	0.070 ± 0.035	0.052	0.040 ± 0.020	0.041 ± 0.009	N.S
**Potassium urinary levels mmol/min ± SD**	0.041 ± 0.024	0.047 ± 0.026	N.S	0.049 ± 0.013	0.041 ± 0.018	N.S	0.033 ± 0.010	0.065 ± 0.039	0.048	0.055 ± 0.038	0.043 ± 0.011	N.S
**Glucose Mmol/min ± SD**	0.0004 ± 0.0003	0.0004 ± 0.0002	N.S	0.0006 ± 0.0002	0.0006 ± 0.0004	N.S	0.0005 ± 0.0002	0.0018 ± 0.0016	N.S	0.0003 ± 0.0002	0.0007 ± 0.0006	N.S

SUPPLEMENTAL TABLE 2. Metabolic measurements for male control and Myh9&10 conditional knockout mice.

	4 Weeks (Pre-Doxycycline)			6 weeks			9 weeks			12 weeks		
	Control n = 10	Myh9; Myh10 cKO n = 7	Sig	Control n = 10	Myh9; Myh10 cKO n = 8	Sig	Control n = 10	Myh9; Myh10 cKO n = 8	Sig	Control n = 6	Myh9; Myh10 cKO n = 7	Sig
**Weight g ± SD**	16.19 ± 2.35	13.62 ± 2.42	0.044	21.45 ± 1.49	20.65 ± 1.70	N.S	24.92 ± 2.71	22.50 ± 1.77	0.045	27.85 ± 4.16	22.56 ± 2.25	0.014
**Water drank ml/g ± SD**	0.39 ± 0.18	0.51 ± 0.17	N.S	0.24 ± 0.08	0.45 ± 0.12	0.003	0.25 ± 0.09	0.47 ± 0.19	0.020	0.30 ± 0.14	0.36 ± 0.06	N.S
**Urine ml/g ± SD**	0.067 ± 0.050	0.074 ± 0.083	N.S	0.040 ± 0.025	0.087 ± 0.054	0.059	0.074 ± 0.050	0.185 ± 0.094	0.020	0.063 ± 0.019	0.107 ± 0.036	N.S
**PH ± SD**	6.35 ± 0.24	6.21 ± 0.27	N.S	6.60 ± 0.39	6.63 ± 0.44	N.S	6.45 ± 0.37	5.38 ± 0.35	<0.001	6.50 ± 0.45	5.07 ± 0.19	<0.001
**Osmolality mmol/kg ± SD**	1014.50 ± 421.46	1042.43 ± 503.67	N.S	1223.40 ± 638.13	1171.38 ± 1452.29	N.S	1119.10 ± 583.62	592.50 ± 324.70	0.029	804.50 ± 349.46	694.57 ± 220.96	N.S
**Total urinary protein Mmol/min ± SD**	0.002 ± 0.0008	0.001 ± 0.0007	N.S	0.006 ± 0.0023	0.005 ± 0.0027	N.S	0.007 ± 0.0032	0.005 ± 0.0032	N.S	0.008 ± 0.0036	0.004 ± 0.0022	0.048
**Sodium urinary levels mmol/min ± SD**	0.042 ± 0.012	0.043 ± 0.022	N.S	0.056 ± 0.021	0.054 ± 0.033	N.S	0.056 ± 0.023	0.036 ± 0.017	0.053	0.049 ± 0.015	0.038 ± 0.014	N.S
**Potassium urinary levels mmol/min ± SD**	0.056 ± 0.026	0.043 ± 0.025	N.S	0.056 ± 0.023	0.051 ± 0.032	N.S	0.057 ± 0.024	0.030 ± 0.017	0.016	0.047 ± 0.019	0.037 ± 0.012	N.S
**Glucose Mmol/min ± SD**	0.0003 ± 0.0001	0.0003 ± 0.0001	N.S	0.0006 ± 0.0003	0.0006 ± 0.0003	N.S	0.0005 ± 0.0003	0.0019 ± 0.0012	0.015	0.0004 ± 0.0001	0.0010 ± 0.0022	0.0002

SUPPLEMENTAL TABLE 3. Serum measurements of control and Myh9&10 conditional knockout mice.

	6 Weeks			9 Weeks			12 Weeks		
	Control n = 13	Myh9; Myh10 cKO n = 10	Sig	Control n = 16	Myh9; Myh10 cKO n = 12	Sig	Control n =12	Myh9; Myh10 cKO n =16	Sig
Glucose mg/dL ± SD	231.15 ± 21.67	231.80 ± 38.80	N.S	218.25 ± 31.20	195.73 ± 36.35	N.S	211.50 ± 38.93	169.31 ± 59.03	0.032
Urea nitrogen mg/dL ± SD	22.23 ± 5.59	21.00 ± 4.88	N.S	23.88 ± 4.10	59.09 ± 42.43	0.020	28.67 ± 4.01	81.44 ± 43.22	<0.001
Creatinine mg/dL ± SD	0.15 ± 0.04	0.17 ± 0.06	N.S	0.140± 0.03	0.32 ± 0.15	0.002	0.15 ± 0.04	0.36 ± 0.14	<0.001
Albumin g/dL ± SD	3.07 ± 0.18	3.09 ± 0.14	N.S	2.96 ± 0.17	3.03 ± 0.31	N.S	3.05 ± 0.27	3.06 ± 0.25	N.S

SUPPLEMENTAL TABLE 4. Antibodies and molecules used for immunohistochemistry and western blot evaluation.

Antibody	Dilution	Catalog number	Company
Myh9	1:2000	M8064-25	Sigma-Aldrich
Myh9	1:500	909801	Biolegend
Myh14	1:500	919201	Biolegend
Myh10	1:500	909901	Biolegend
Fluorescein-LTL	1:200	FL-1321	Vector Laboratories
Calbindin D28K	1:1000	NBP2-50028	Novus Biologicals
Megalin (Lrp2)	1:200	Ab76969	Abcam
Sglt2	1:200	Sc-47402	Santa Cruz Biotechnology
UMOD (human)	1:200	PA5-46959	Thermo Scientific
UMOD (mouse)	1:200	PA5-47706	Thermo Scientific
NogoA (RTN4)	1:500	AHP1799	Biorad
Aqp2	1:200	SC-9882	Santa Cruz Biotechnology
Gapdh	1:4000	AM4300	Thermo Scientific
Calreticulin	1:1000	Ab92516	Abcam
NKCC2 – T9	2ug	T9	Developmental Hybridoma
NHE3	1:200	-	McDonough Laboratory
NHE3	1:100	SC-136368	Santa Cruz Biotechnology
XBP1	1:500	Ab85546	Abcam
Na+ K+ Atpase	2ug	A5	Developmental Hybridoma
p-SLC12A3 (pNCC)	1:500	NBP2-60775	Novus Biologicals
Romk1	1:200	APC-001	Alomone Labs
Villin488	1:100	SC-58897 AF488	Santa Cruz Biotechnology
ATF-6alpha	1:2000	Sc-166659	Santa Cruz Biotechnology
Alpha-Tubulin	1:10000	2144	Cell Signaling Technology
TFRC	1:1000	Ab84036	Abcam
Wheat Germ Agglutinin (WGA)Oregon Green488	1:200	W6748	Invitrogen
Calnexin	1:200; 1:10000	PA5-34754	Invitrogen
Hoechst 33342 (DAPI)	1:5000	#62249	Thermo Scientific
Kim1	1:1000	AF1817-SP	R&D Systems
CD3	1:50	Ab16669	Abcam
F4/80	1:100	NB600-404	Novus Biologicals

Table 5. Secondary antibodies used for immunohistochemistry

Antibody	Catalog number	Company
Cy3 AffiniPure Donkey anti-rabbit	711-165-152	Jackson ImmunoResearch
Cy3 AffiniPure Donkey anti-rat	712-165-150	Jackson ImmunoResearch
Cy3 AffiniPure Donkey anti-goat	705-165-147	Jackson ImmunoResearch
Cy3 AffiniPure Donkey anti-sheep	713-165-147	Jackson ImmunoResearch
Cy3 AffiniPure Donkey anti-mouse	715-165-150	Jackson ImmunoResearch
Alexa Fluor 488 AffiniPure Donkey anti-goat	705-545-147	Jackson ImmunoResearch
Alexa Fluor 488 AffiniPure Donkey anti-Mouse	715-545-150	Jackson ImmunoResearch
Alexa Fluor 488 AffiniPure Donkey anti-chicken	703-545-155	Jackson ImmunoResearch
Alexa Fluor 488 AffiniPure Donkey anti-sheep	713-545-003	Jackson ImmunoResearch
Donkey anti-Ovine Alexa Fluor 488	A11015	Invitrogen
Donkey anti-goat Alexa Fluor 647	A21447	Invitrogen
Horse anti-mouse DyLight 549	DI2549	Vector Labs

Supplemental Figures

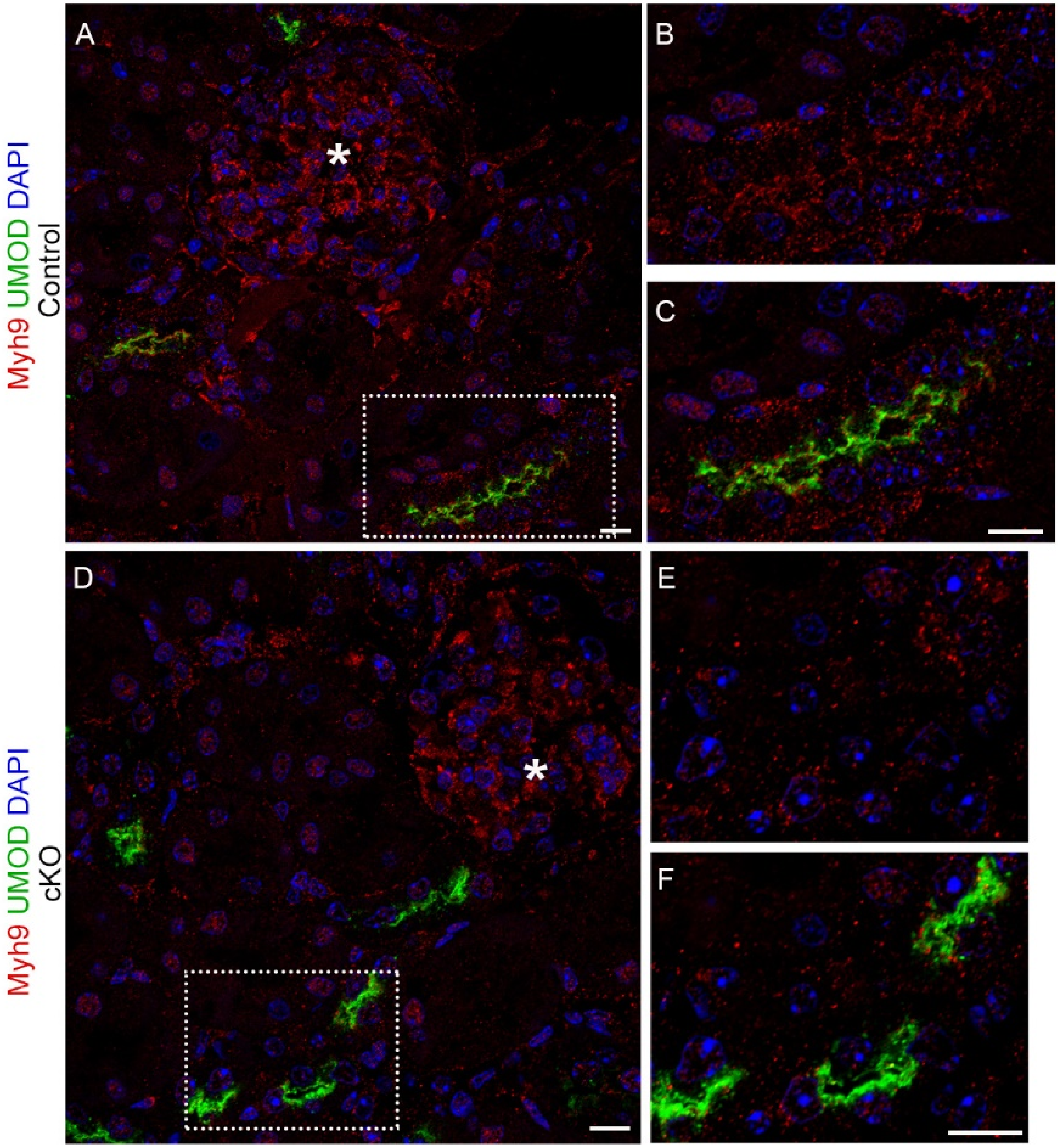

Figure S1: Loss of MYH9 protein in the thick ascending limb of renal tubules in *Myh9&10* cKO mice is evident by 6 weeks of age. A-C: 6-week-old kidneys from control and cKO mice were co-stained for MYH9, UMOD and DAPI. **A-C:** MYH9 (red) is expressed within the glomerulus (A, asterisks) and the TAL segment of the loop of Henle (uromodulin-positive, green) in control mouse kidneys. **D-F:** Genetic inactivation of *Myh9* in the renal tubules utilizing the *Pax8->rtTA; Tet-O->cre* system leads to loss of MYH9 expression in the TAL segment (D-F), but not in the glomerulus (D, asterisk). Boxed regions in the control (A) and cKO (D) are enlarged to show loss of MYH9 expression in the cKO (E, F) compared to the control tubules (B, C). Scale=10μm. Images are representative of at least three control and three cKO kidneys sectioned and stained.

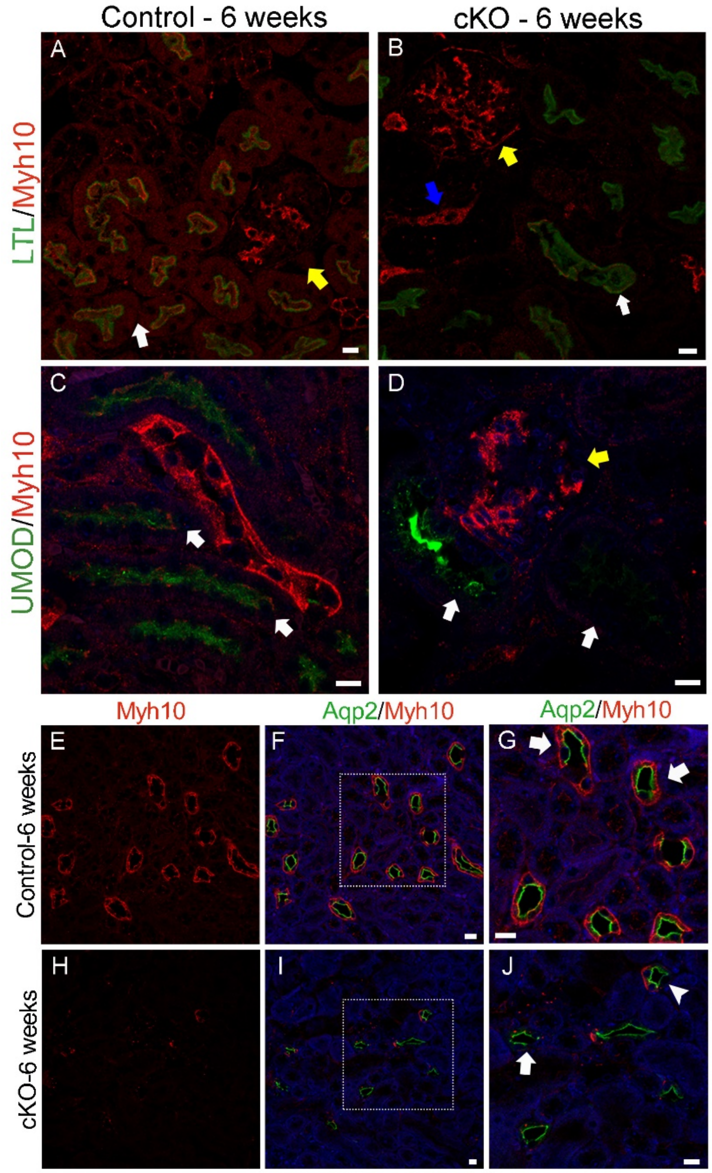

Figure S2: Loss of MYH10 protein in renal tubular segments of *Myh9&10* cKO mice is evident by 6 weeks of age. A-J: Representative images from 6-week old control and cKO mouse kidney sections were stained to assess the expression pattern of MYH10 protein in proximal tubule, thick ascending limb and collecting duct segments. **A-B:** Control (A) and cKO (B) kidneys co-stained for proximal tubular marker (FITC-LTL) and MYH10 show MYH10 staining (red) in control sections alongside the LTL stained (green) structures in proximal tubules as well as in intracellular regions (white arrow). In cKO kidney sections at 6-weeks, loss of MYH10 is variable with some tubules (white arrow) maintaining minimal expression of MYH10 while others show a complete loss of intracellular and apical staining. The *Pax8->rtTA; Tet-O->cre* system does not target the glomeruli (A, B, D yellow arrow), therefore, normal MYH10 expression is observed in the glomeruli of cKO kidneys. Infiltrating cells (B, blue arrow) in cKO kidneys also express MYH10 (red). **C-D:** Control (C) and cKO (D) kidneys co-stained for TAL marker UMOD and MYH10 show MYH10 staining (red) in control sections along the apical membrane in UMOD (green) positive tubules (white arrows). cKO kidneys show TAL tubules with abnormal UMOD (green) localization that are devoid of MYH10 (red) expression (white arrow). **E-J:** Principal cells of the collecting duct

have the highest level of MYH10 expression in the kidney. Control kidneys co-stained for collecting duct specific marker, aquaporin-2 (AQP2) and MYH10 show expression of MYH10 (red) in the AQP2-positive (green) cells (E, F, G, arrow) (white box region enlarged in G). cKO kidneys show complete loss of MYH10 from some AQP2-positive tubules (white arrow), while other tubules maintain minimal expression of MYH10 (white arrowhead) (H, I, J). Scale=10μm. Images are representative of at least three control and three cKO kidneys sectioned and stained.

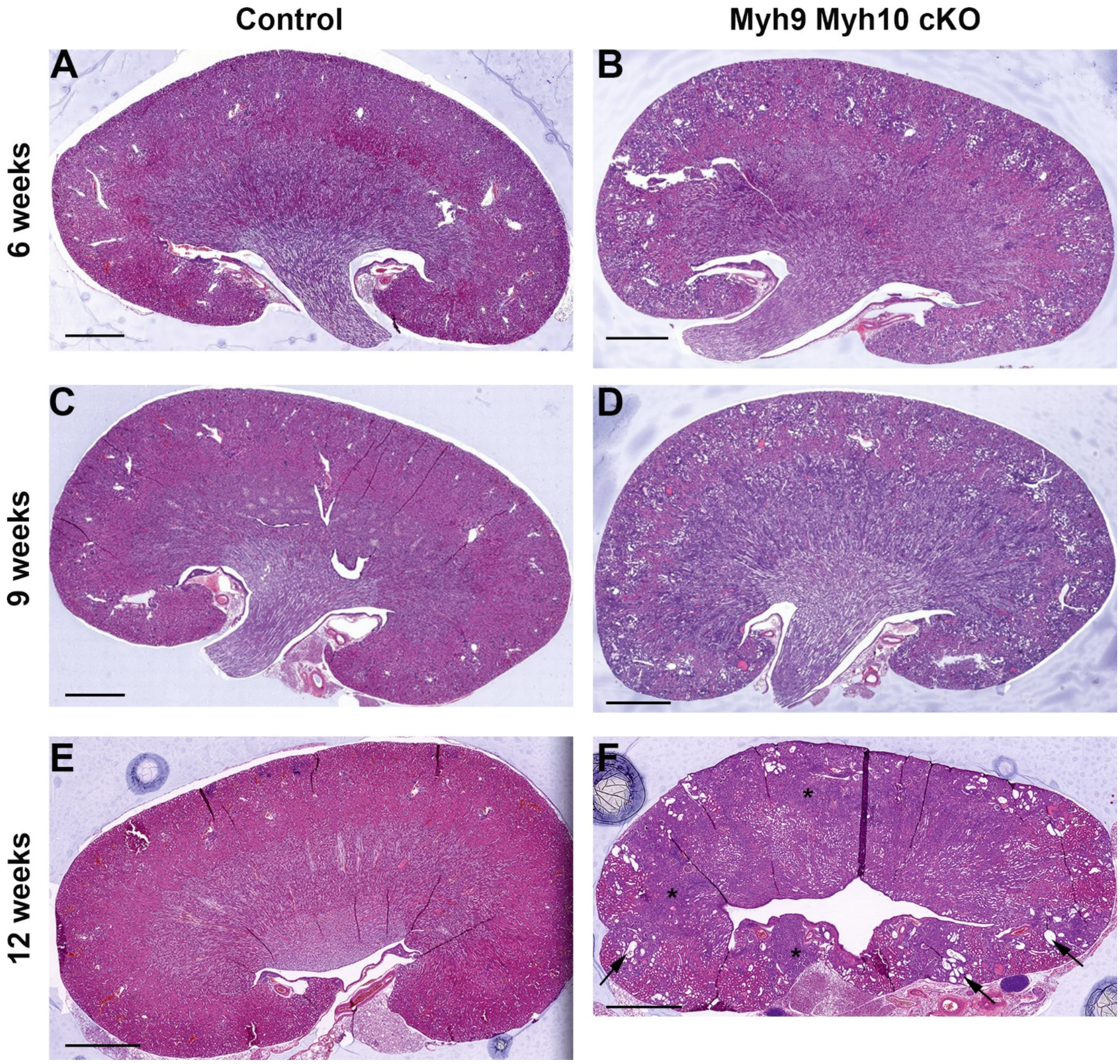

Figure S3: Cellular infiltration and tubular dilation in *Myh9&10* cKO mice start as focal areas in the cortex and progress into the medullary region. A-F: Images of hematoxylin and eosin stained control and cKO kidneys show progression of dilation and hypercellularity. **A-B:** At 6-weeks of age mild dilation in the cortical region is observable in the cKO mice (B) compared to littermate controls (A). **C-D:** At 9-weeks of age cortical dilation and hypercellularity is noticeable in the cortex and cortico-medullary regions in cKO mice (D) compared to littermate controls (C). **E-F:** At 12-weeks, extensive tubular dilation (arrows), and regions of infiltrating cells (asterisks) are observed throughout the kidneys of the cKO mice (F) compared to the kidneys of the control littermates (E). Scale bars =1mm. Images are representative of at least three control and 3 cKO kidneys sectioned and stained.

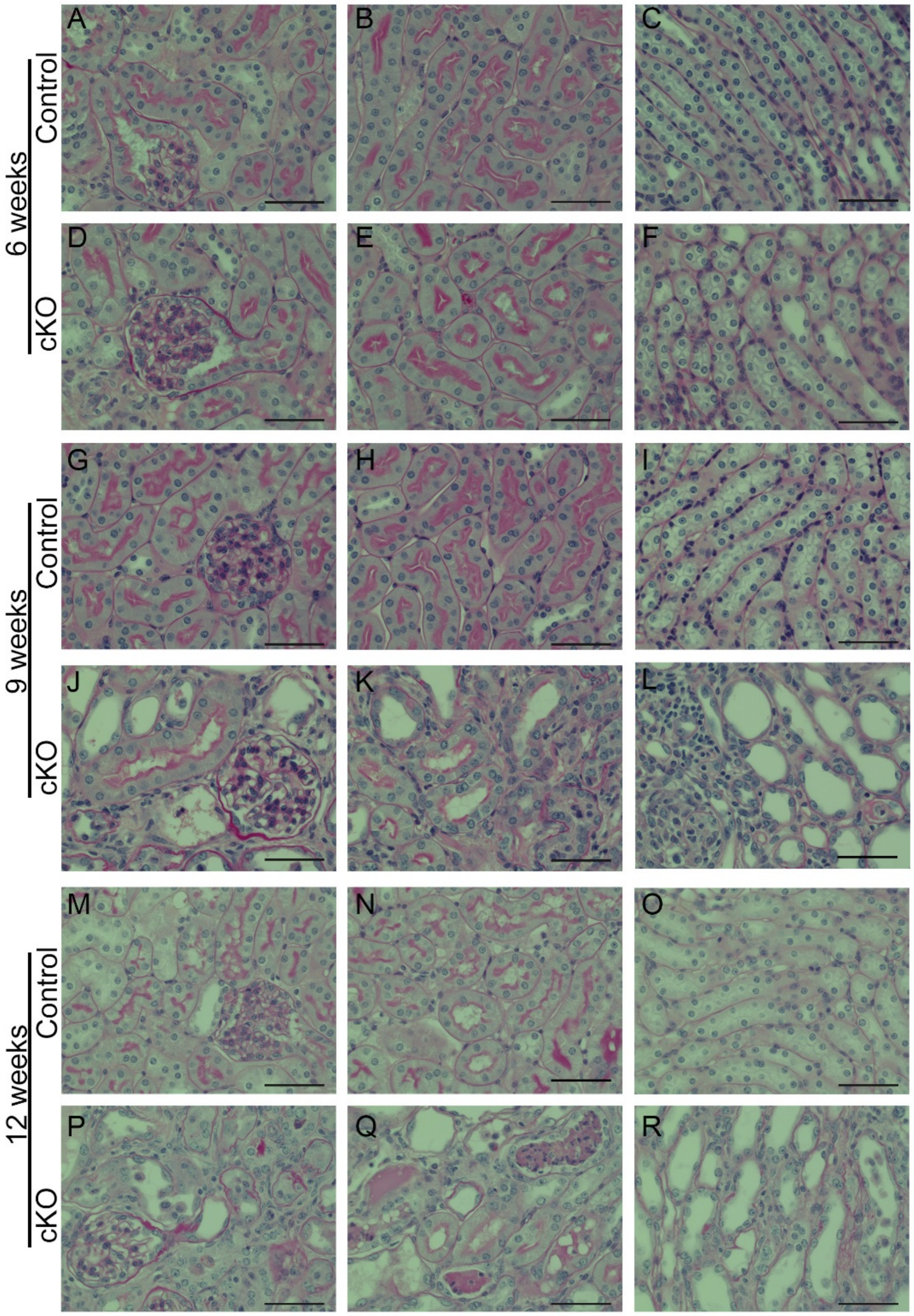

Figure S4: Periodic-Acid Schiff staining shows tubular dilation and loss of brush borders with disease progression in the *Myh9&10* cKO kidneys. **A-R:** Periodic Acid Schiff staining was used to identify changes in the brush borders of the renal tubules. **A-F:** At 6-weeks of age the conditional knockout kidneys show minor loss of the brush border (D-F) compared to littermate controls (A-C). **G-L:** By 9-weeks of age, loss of the brush border is evident with entire cells missing the brush borders on the apical membrane (J-L) compared to littermate controls (G-I). **M-R:** At 12-weeks of age damage to tubules is extensive as evidenced by dilated tubules with cellular debris in the lumen (P-R) compared to littermate controls (M-O). Scale bars = 50μm. Images are representative of at least three control and three cKO kidneys sectioned and stained.

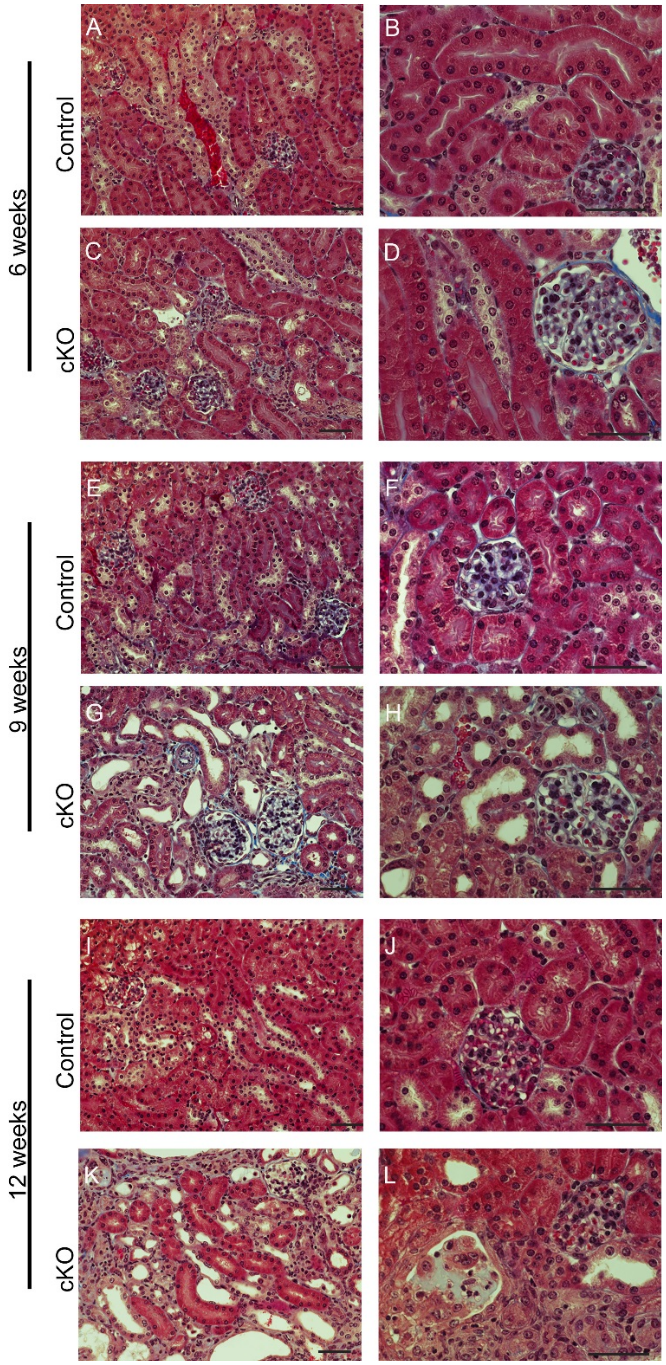

Figure S5: Trichrome staining shows mild fibrosis with the progression of kidney disease in *Myh9&10* cKO mice. A-L: Trichrome staining was used to determine if the progression of kidney disease in the conditional knockout mice involved fibrosis. **A-D:** At 6-weeks of age, the littermate controls (A,B) were indistinguishable from the cKO kidneys (C,D). **E-H:** At 9-weeks of age focal regions of mild fibrosis (light blue staining) are evident in the cKO kidneys (G,H) compared to littermate controls (E,F). **I-L:** At 12-weeks of age major structural changes are observed in the cKO kidneys (K,L) compared to littermate controls (I,J); however, fibrosis remains mild despite the major structural changes. Scale bars = 50μm. Images are representative of at least three control and three cKO kidneys sectioned and stained.

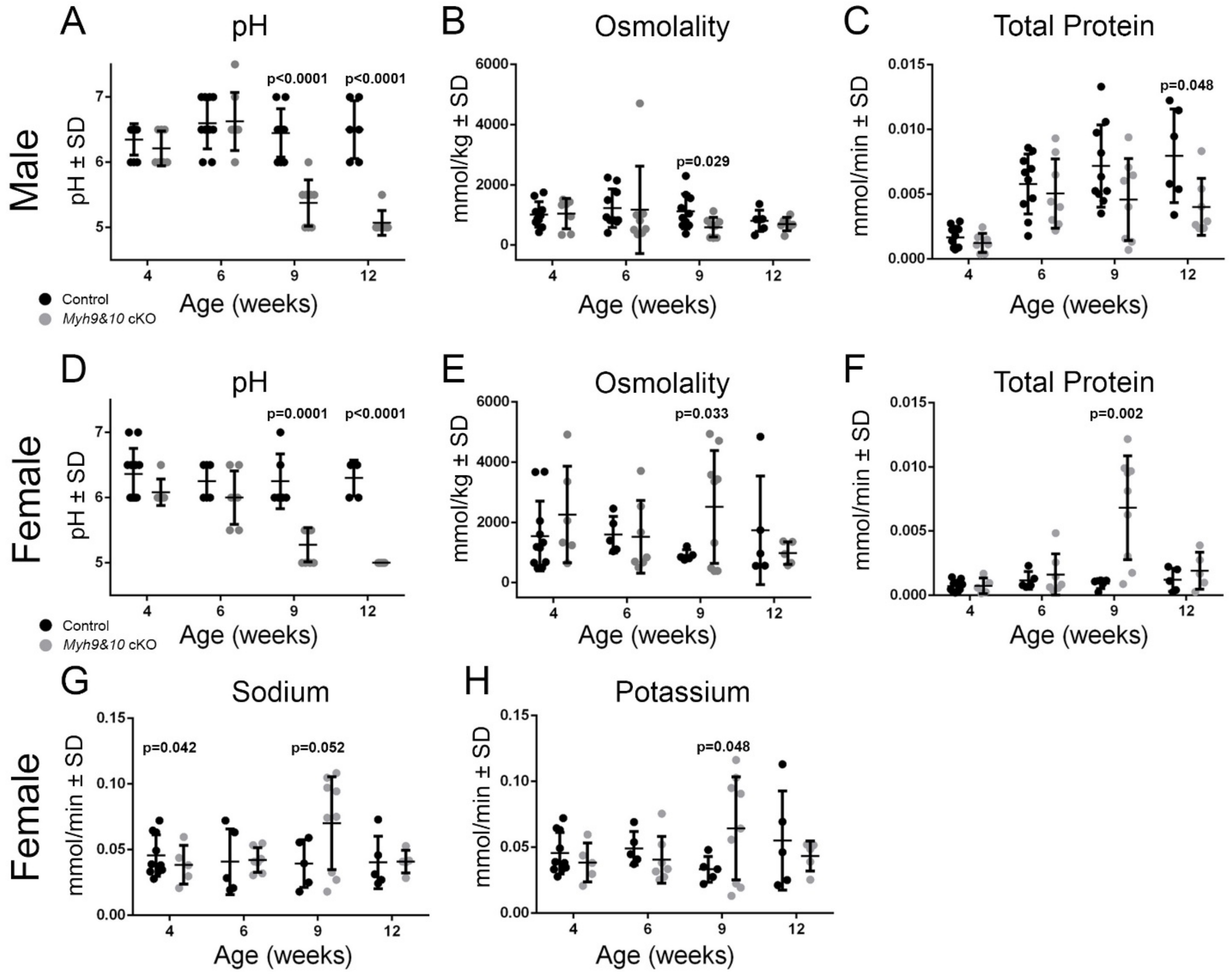

Figure S6: Urine Chemistry highlights the decline in kidney function in *Myh9&10* cKO mice. A-H: Urine collected from control (black) and cKO (gray) mice was analyzed for urinary pH (A,D), osmolality (B,E) and total urinary protein (C,F) as well as urinary sodium (G) and potassium (H). **A-C**: In male mice, loss of MYH9&10 leads to reduced urinary pH (A) and a decrease in both urinary osmolality (B) and total protein (C) at 9-weeks of age compared to the littermate controls. **D-F:** Loss of MYH9&10 in female mice also leads to reduced urinary pH (D), but an increase in both urine osmolality (E) and total urinary protein (F) at 9-weeks of age. **G-H:** In female mice, loss of MYH9&10 lead to increased urinary excretion of sodium and potassium mimicking a salt wasting phenotype at 9-weeks of age. Sample sizes were as follows for females: 4 weeks n=12 and 8, 6 weeks n=14 and 10, 9 weeks n=7 and 9, 12 weeks n=5 and 5 for control and cKO females, respectfully. Sample sizes for males were as follows: 4 weeks n=10 and 7, 6 weeks n=10 and 8, 9 weeks n=10 and 8, 12 weeks n=6 and 7 for control and cKO males, respectfully. SAS software suite developed for multivariate analysis was used for statistics.

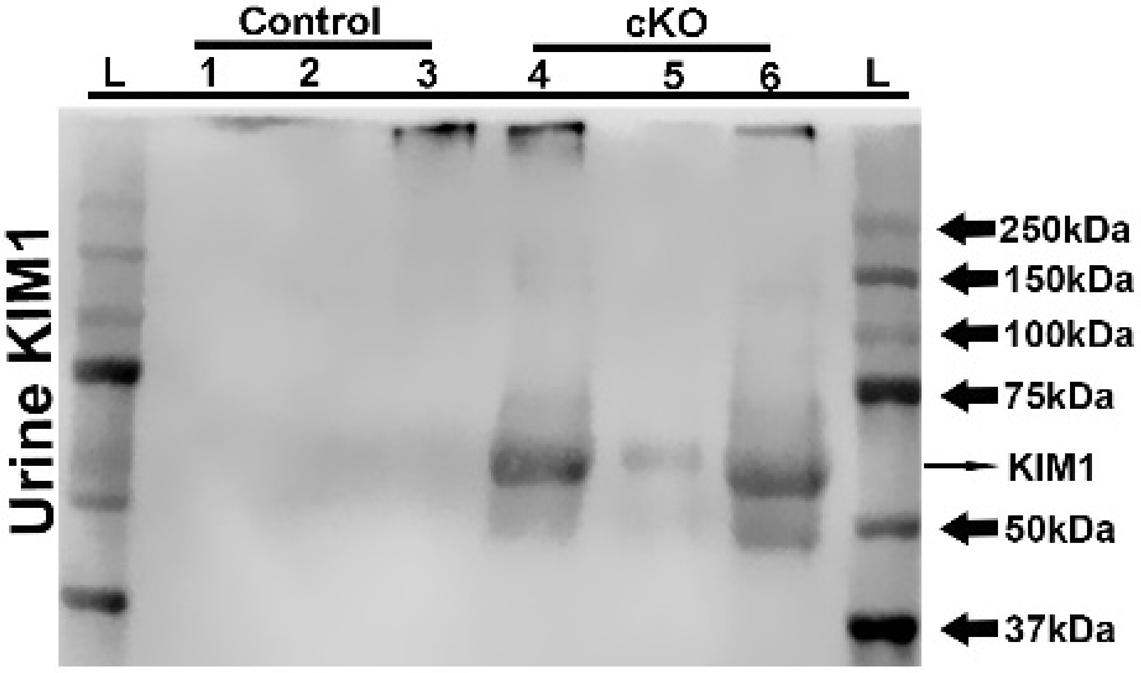

Figure S7: KIM1 is excreted in the urine of *Myh9&10* cKO mice. Urine collected from 9-week old control and cKO male mice was probed for KIM1 excretion. KIM1 was not detected in the urine of control mice (Lanes 1, 2 &3). KIM1 was detected in the urine of cKO mice (Lanes 4, 5 &6). n=3 for control and cKO samples.

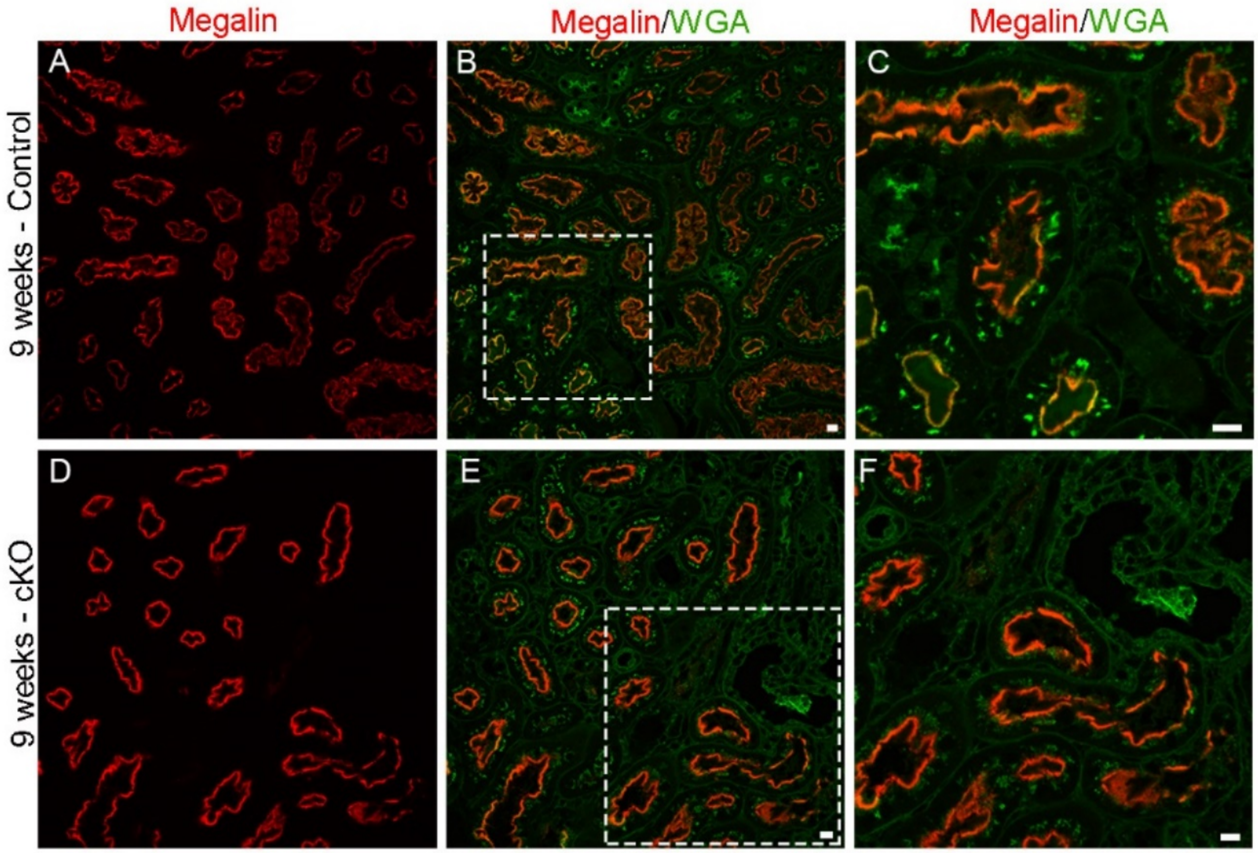

Figure S8: Inactivation of *Myh9&10* in mouse renal tubules does not affect localization of proximal tubule specific receptor, megalin. A-F: To evaluate the impact of loss of *Myh9* and *Myh10* on the proximal tubule specific receptor, megalin, 9-week-old kidneys were stained for megalin and WGA. **A-C:** Control kidney sections show proximal tubules with megalin (red) localized to the apical membrane with WGA (green). **D-F:** cKO kidney sections also show megalin (red) staining on the apical membrane. Boxed regions in B and E are magnified in the adjacent images. Scale bars=10µm. Images are representative of at least three control and three cKO kidneys sectioned and stained.

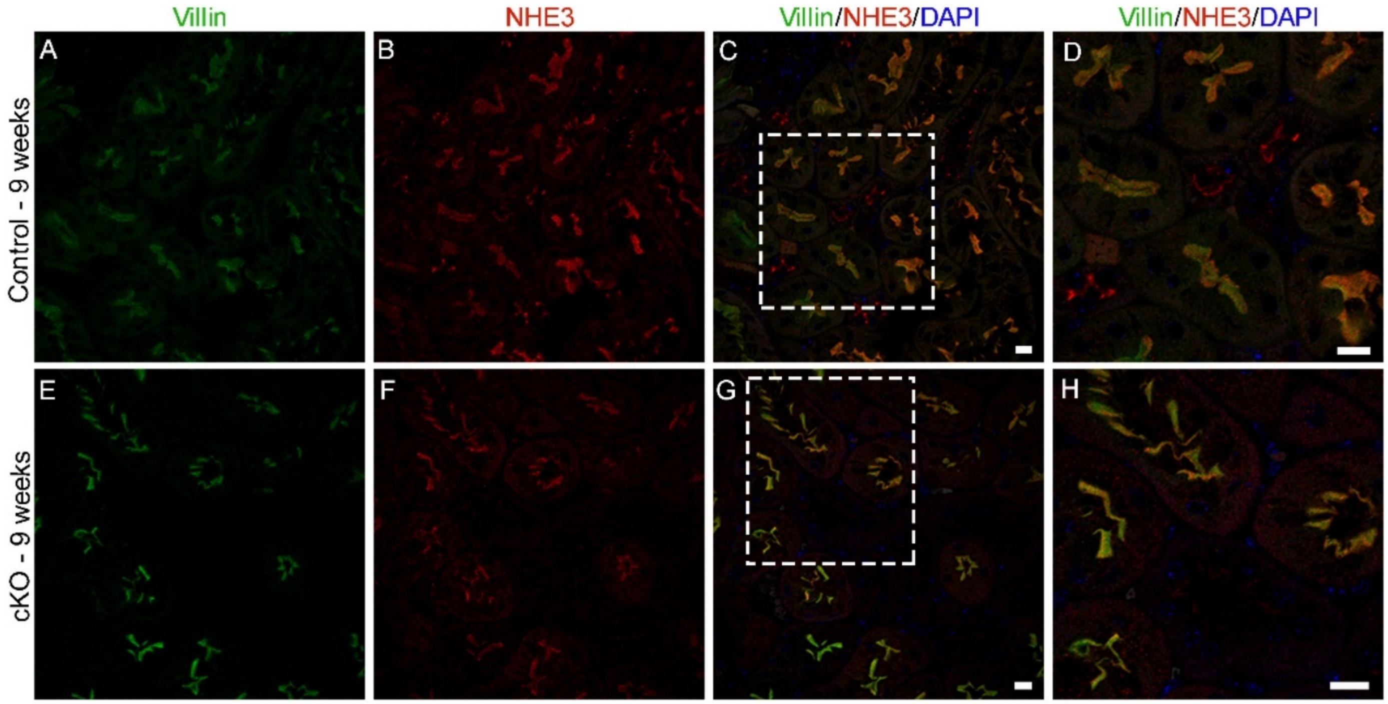

Figure S9: Loss of MYH9&10 leads to mild decrease in NHE3 expression in the proximal tubules. A-H: Images from control and cKO kidney sections of 9-week old cohorts were stained for brush border protein, villin and NHE3 transporter. **A-D:** Control kidneys show several proximal tubule brush borders stained with Villin (green) that colocalize with NHE3 (red) to varying levels. **E-H:** cKO kidney sections show several dilated proximal tubules that show colocalization of NHE3 with villin. Reduction in NHE3 staining intensity is apparent in cKO tubules (F, G, H). Boxed regions in C and G are enlarged in the adjacent images. Scale=10μm. Images are representative of at least three control and three cKO kidneys sectioned and stained.

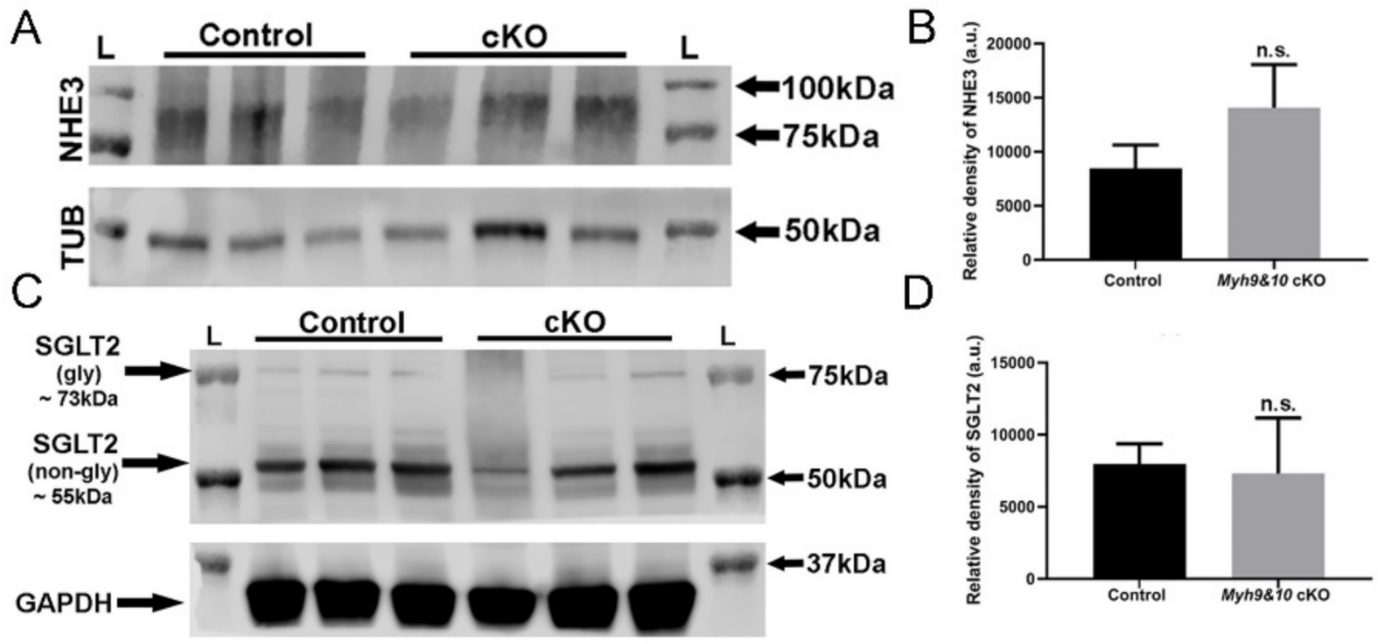

Figure S10: Expression of proximal tubule sodium cotransporters, NHE3 and SGLT2, does not change in *Myh9&10* cKO kidneys. A: Whole kidney lysates of cKO and control mice from 9-week cohorts were subjected to immunoblot analysis to detect protein expression levels of NHE3 and tubulin. Immunoblot analysis detected a ~90kDa band for NHE3 in control and cKO kidneys. Intensity of the NHE3 bands did not show any major changes in 9-week cKO samples compared to the controls. **B:** The bar graph shows the quantification of the relative density of the NHE3 bands and no significant change in NHE3 protein expression between the cKO and control kidneys (p value=0.09 by 2-tailed t-test, n=3 for controls and cKO). **C:** Whole kidney lysates of cKO and control mice from 9-week cohorts were subjected to immunoblot analysis to detect protein expression levels of SGLT2 and GAPDH. Immunoblot analysis detected two bands, a ~55kDa band that corresponds to non-glycosylated SGLT2 and a ~73kDa band that corresponds to glycosylated SGLT2 in control and cKO kidneys. Intensity of the SGLT2 bands did show sample dependent variation. **D:** The bar graph shows the quantification of the relative density of the SGLT2 bands and no statistically significant differences in SGLT2 expression between the control and cKO kidneys (p value=0.8 by 2-tailed t-test; n=3 for controls and cKO). L indicates ladder lanes and molecular weight (kDa) corresponds to the ladder bands. Error bars depict standard deviation.

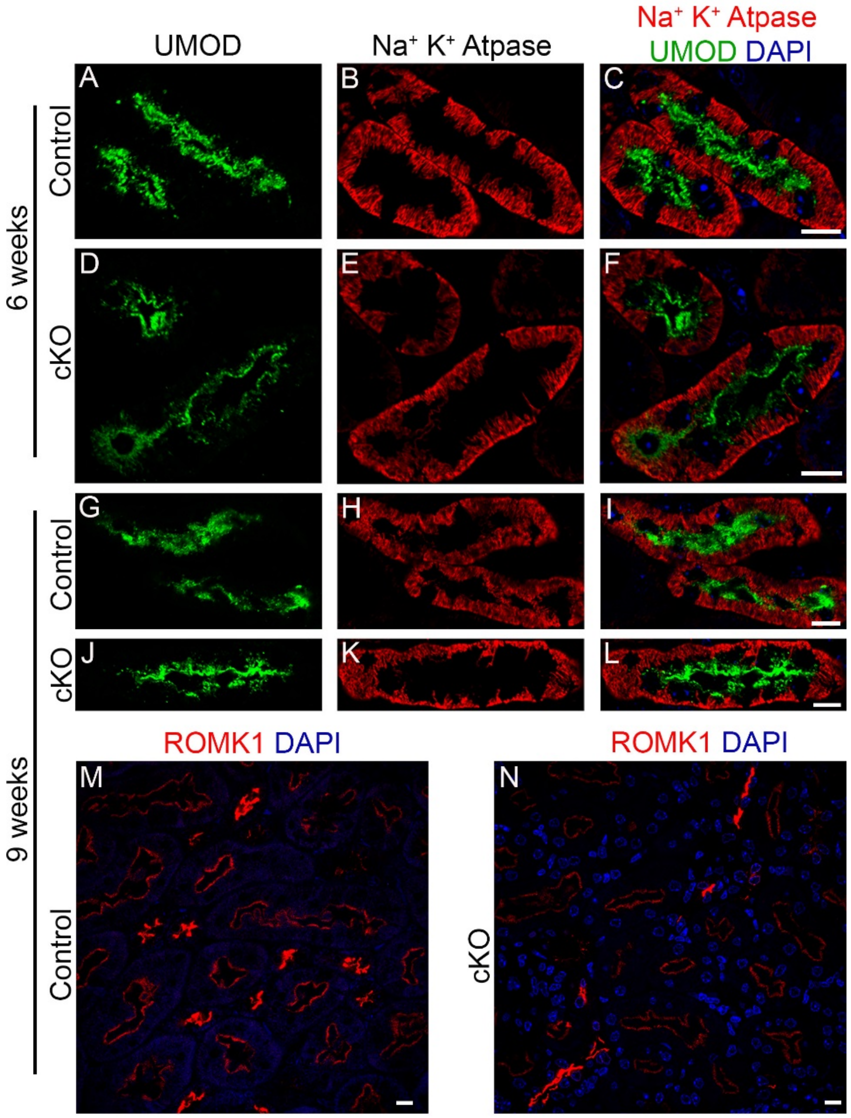

Figure S11: Sodium potassium pump Na$^+$K$^+$ Atpase and the inward rectifying potassium channel ROMK1 localization along the TAL segment is not affected by loss of MYH9&10 proteins. A-N: Further characterization of changes to the TAL segment in *Myh9&10* cKO mice included immunofluorescence for the additional channels and pumps, Na$^+$K$^+$ Atpase and ROMK1. **A-F:** At 6-weeks of age, Na$^+$K$^+$ Atpase (red) is localized to the basal membrane in the cKO (E, F) similar to the control (B, C). UMOD (green) localization in the cKO TAL tubule (D, F) is affected compared to the control TAL segment (A, C). **G-L:** In the cKO kidneys at 9-weeks of age Na$^+$K$^+$ Atpase localizes to the basal membrane but mild thinning is observed (K, L) as compared to the control (H, I). UMOD localization to the apical membrane is decreased in the cKO (J, L) as compared to the control tubules (G, I). **M-N:** ROMK1 (red) localization in the TAL segment is not affected in the 9-week old cKO tubules (N) compared to littermate control mice (M). Scale bars = 10μm for A-L and 50μm in M, N. Images are representative of three control and three cKO kidneys sectioned and stained.

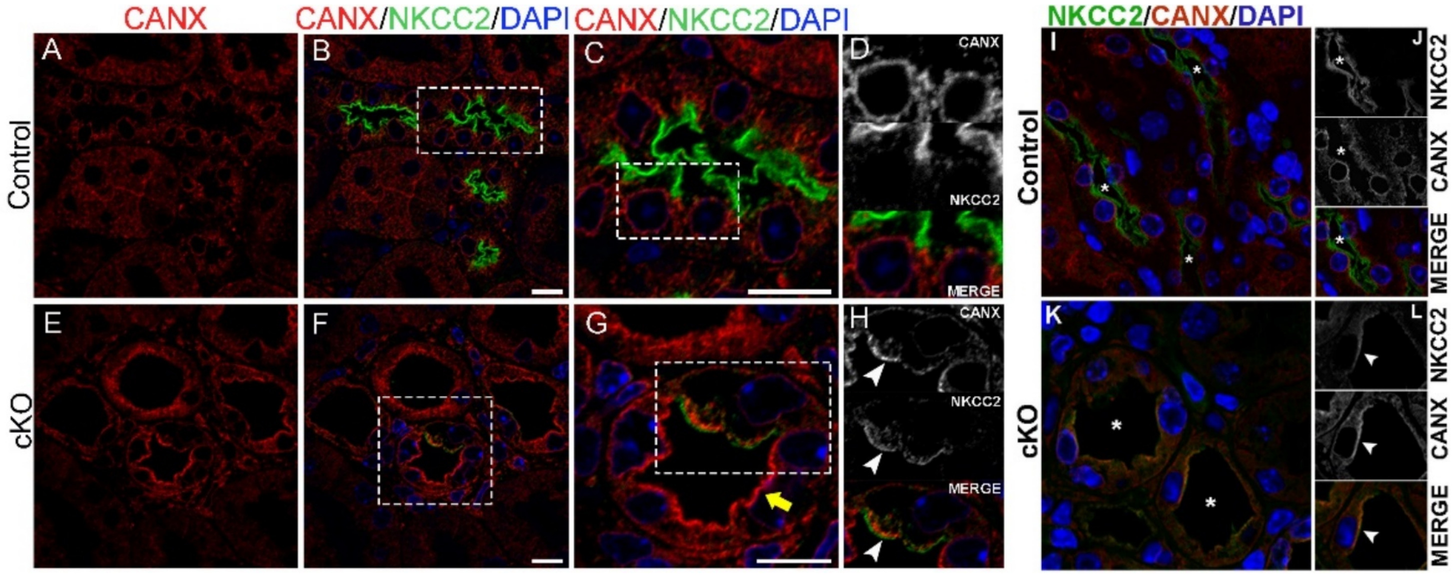

Figure S12: Mislocalization of ER chaperone protein calnexin is observed in *Myh9&10* cKO TAL cells. A-H: 9-week old Bouin's fixed control and cKO kidney sections stained to visualize ER chaperone protein, CANX and NKCC2 in the TAL tubules. **A-B:** Tubules from control kidneys show the expression and localization pattern of CANX (red, A) relative to NKCC2 (green, B)**. C-D:** Magnified images from representative regions (white boxes in B, C) show that in control TAL segments NKCC2 localizes to the apical membrane, while CANX localizes to the nuclear envelope as well as intracellular punctate structures. **E-G:** Dilated TAL tubules from cKO kidneys show partial loss of NKCC2 (white box, F, G) while CANX localizes to subapical regions in the dilated tubule (yellow arrow). **H:** Magnified images (white box in G) demonstrate partial colocalization between CANX and NKCC2 in the subapical regions of the TAL epithelium of the cKO kidney sections (white arrowheads) **I-L:** Images represent 9-week old PFA-fixed control and cKO kidney sections stained to visualize CANX, NKCC2 and DAPI in the TAL tubules. **I-J:** In control kidney sections, NKCC2 (green) localizes to the apical membrane and CANX (red) to the nuclear envelope and to intracellular punctate structures. **K-L:** In cKO kidney sections, CANX localizes to the apical/subapical regions of the dilated tubules (white arrowhead). Individual insets in gray scale along with the merged image clearly depict the colocalization between CANX and NKCC2 inside the TAL cells. Asterisks (*) in all images denotes the lumen. Scale=10μm. Images are representative of at least three control and three cKO kidneys sectioned and stained.

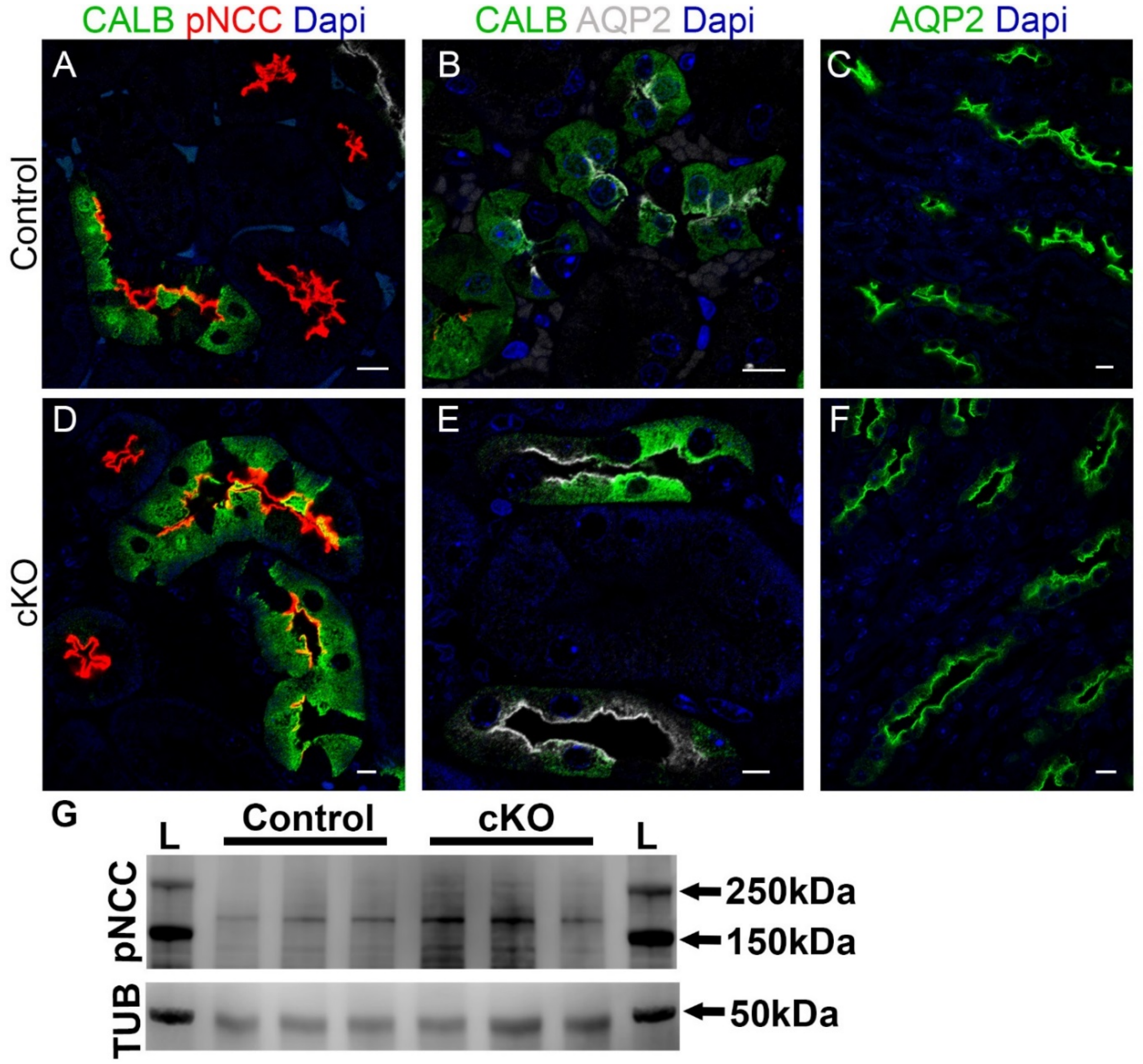

Figure S13: Inactivation of *Myh9&10* does not lead to changes in localization of the distal nephron transport proteins. A-F: Immunofluorescence for calbindin (CALB), phosphorylated sodium chloride cotransporter (pNCC), aquaporin-2 (AQP2) and DAPI were used to determine the impact of MYH9 and MYH10 loss on the distal nephron and collecting duct at 9-weeks of age. **A and D:** Distal tubules expressing CALB (green), pNCC (red) and DAPI are dilated, but the proteins localize properly to the apical membrane in the cKO (D) compared to the control (A). **B and E:** CALB-positive (green) and AQP2-positive (white) connecting segment tubules are also dilated in the cKO (E) compared to the control (B), but apical proteins localize normally. **C and F**: The AQP2-positive (green) collecting duct tubules are also dilated in the cKO (F) compared to the control (C), but AQP2 localizes normally to the apical membrane. Scale =10μm. Images are representative of three control and three cKO kidneys sectioned and stained. **G:** Whole kidney lysates of three cKO and control mice from 9-week cohorts were subjected to immunoblot analysis to detect protein expression levels of pNCC and tubulin. Immunoblot analysis detected a ~160kDa band for pNCC in control and cKO kidneys. Intensity of the pNCC bands were slightly increased in two of the 9-week cKO samples. L indicates ladder lanes and molecular weight (kDa) corresponds to the ladder bands.

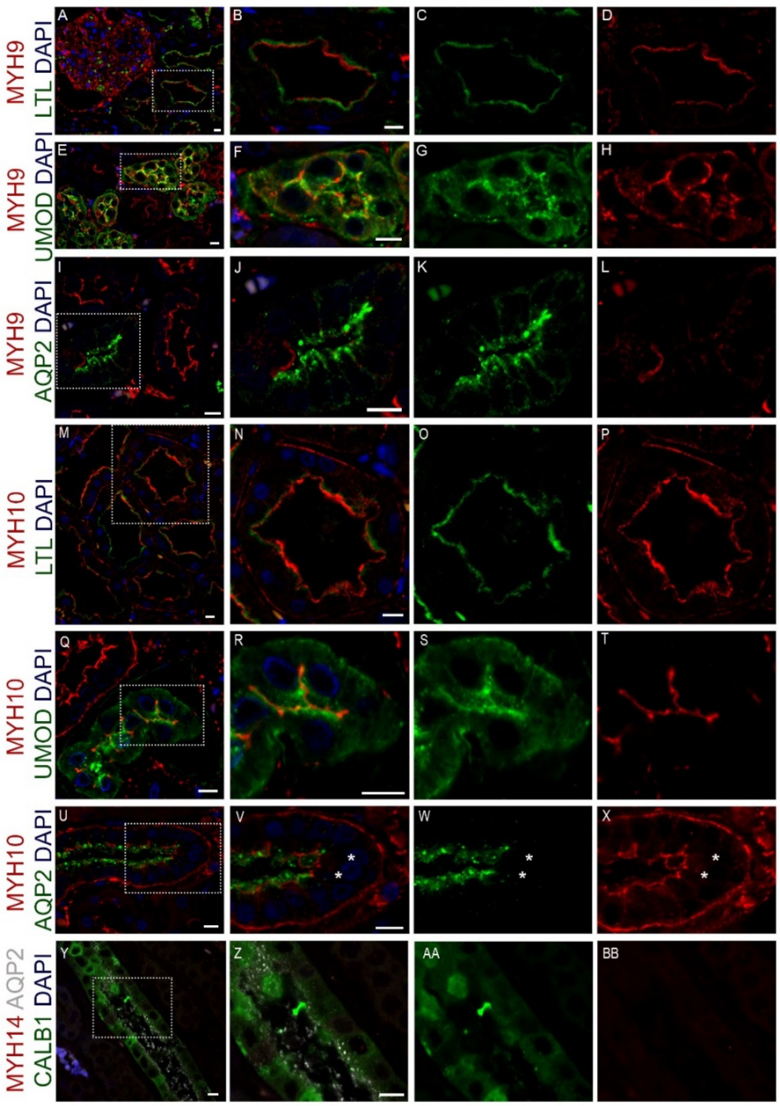

Figure S14: MYH9 and MYH10 proteins localize along the apical and basolateral membranes of renal tubules in human kidneys. A-D: PCTs stained using *Lotus tetragonolobus lectin* (LTL, green) show localization of MYH9 (red) along the apical membrane **E-H:** Apical and basolateral localization of MYH9 (red) is observed in the TAL segments of the loop of Henle, which were identified with co-staining for UMOD (green). **I-L:** MYH9 (red) localizes to the apical membrane of intercalated cells that lack AQP2 (green) expression. **M-P:** Co-staining with LTL (green) shows MYH10 (red) localization along the apical and basal membranes in PCTs. **Q-T:** UMOD-positive (green) TAL tubules show localization of MYH10 (red) on the apical membranes. **U-X:** MYH10 (red) localizes to both the apical and basolateral membranes of AQP2-positive (green) principal cells. Intercalated cells that do not express AQP2 do not show any MYH10 expression (asterisks). **Y-BB:** MYH14 is not expressed in the human kidney. The boxes mark the enlarged regions shown in the adjacent panels. Scale bars = 10μm. Images were obtained from a single human donor kidney.

Supplemental Movie 1: Airy scan z projection of a control kidney section show several tubules with apical membrane localization of UMOD (green) and NKCC2 (red). We also observe colocalization of both proteins along several regions of the apical membrane.

Supplemental Movie 2: Airy scan z projection of a *Myh9&10* cKO kidney section show two dilated tubules with UMOD accumulation in the lumen and loss of NKCC2 from the apical membrane, several intracellular puncta of NKCC2 are observed in some cells.

Supplemental Movie 3: Airy scan z projection of a control kidney section show several tubules with apical membrane localization of UMOD (green) and intracellular staining for Reticulon 4 as thin filaments and along the subapical regions of the cell (RTN-4). Two tubules show RTN-4 staining along the brushborders.

Supplemental Movie 4: Airy scan z projection of a PFA-fixed *Myh9&10* cKO kidney section show a single dilated tubule with increased intracellular UMOD (green) and Reticulon-4 (red) expression. Parts of other tubules are visible that show increase in UMOD and/or RTN4 expression.

Supplemental Movie 5: Airy scan z projection of a PFA-fixed control kidney section shows several tubules with apical membrane localization of UMOD (green) along with some intracellular UMOD. Calreticulin (red) appears as intracellular punctate structures within the cells.

Supplemental Movie 6: Airy scan z projection of a PFA-fixed *Myh9&10* cKO kidney section shows a dilated tubule with increased intracellular expression of UMOD (green) and calreticulin (red) in cells.

Uncropped western blots (raw data) for review:

We perform quantitative western blots using the same PVDF membrane (gel) and probe for protein of interest (UMOD, NKCC2, Calreticulin, ATF6 and XBP1etc) and loading control (Tubulin, GAPDH, TFRC). The images below are single exported image saved directly from the LiCor imager software. As stated in methods section equal amount of protein is loaded from each sample calculated based on quantification data from Pierce (23227) kit.

Ladder used in blots is precision plus protein ladder from BioRad (250kDa, 150kDa, 100kDa, 75kDa, 50kDa, 37kDa, 25kDa, 20kDa, 15kDa and 10kDa). Blots were developed using Licor Odyssey, exposed for 30sec at 700nm and 30 sec up to 2min in chemiluminescence channel. Ladders were visible in the chemiluminescence channel in some blots, in other blots the 700nm and chemilumiscence channels were merged to represent the ladders clearly.

6 weeks UMOD blot in Figure 4M

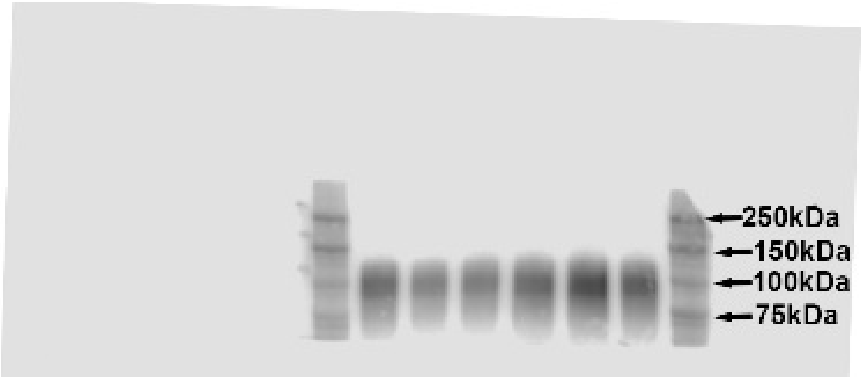

6 weeks UMOD blot loading control (tubulin). Membrane was cut between 75kDa and 50kDa ladder bands. The top bands are the predicted 55kDa mol.wt band for tubulin. The lower bands are closer to 40kDa non-specific bands that appear at varying intensities with the tubulin antibody.

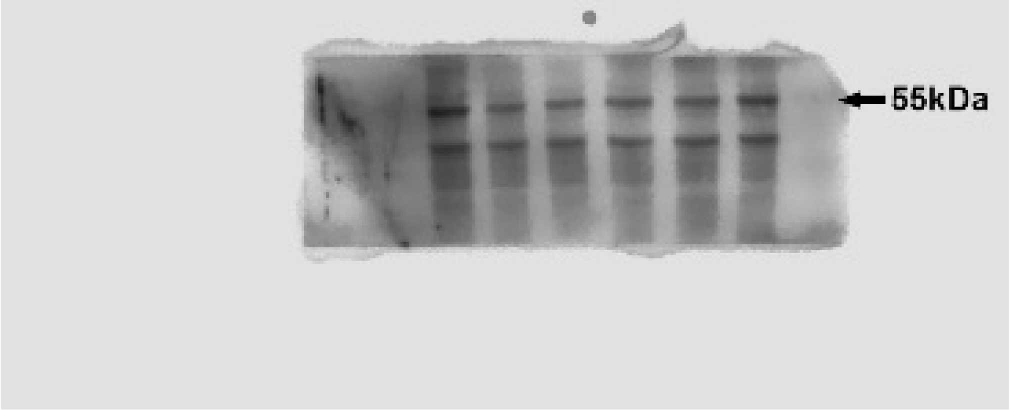

Figure 4N

9 weeks UMOD blot show the UMOD between 100kDa and 75kDa ladder bands on either sides. These bands are very consistent between all samples tested. Two ladder lanes, one on each side.

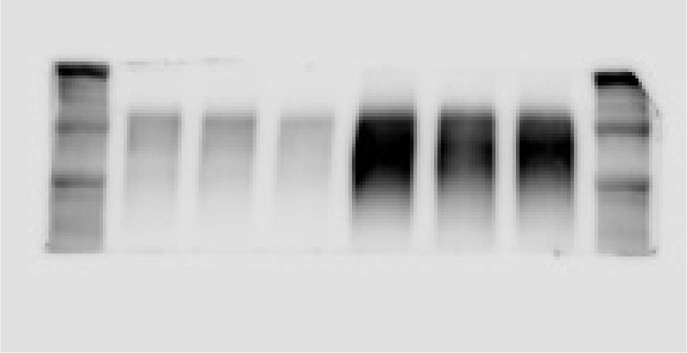

9 week tubulin band (loading control) for the UMOD blot – Figure 4N

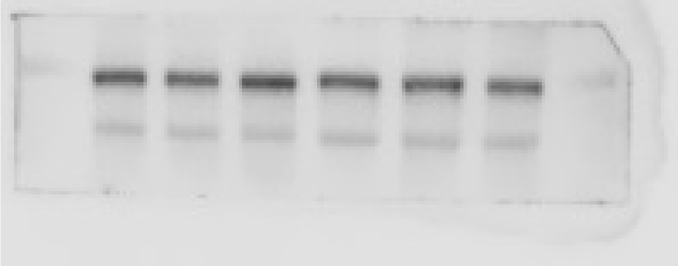

Figure 4O

12 week UMOD blot – Three ladder lanes, two ladder lanes on the right side near the cKO samples.

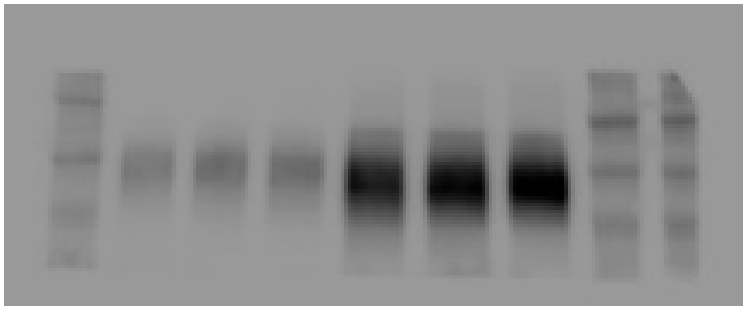

12 week tubulin band for UMOD blot – Figure 4O

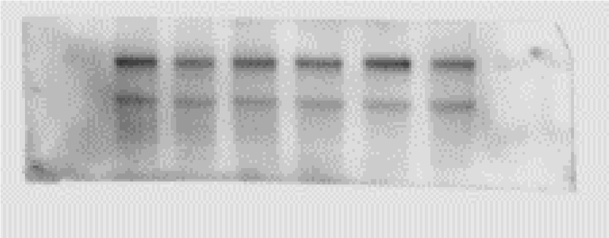

Figure 4Q – PNGASE F blot has the complete gel represented in the figure.

Figure 5M

6 week NKCC2 blot.

Consistent bands little above 150kDa ladder bands (predicted mol.wt for NKCC2). Also a consistent band with a varying intensity lower than 75kDa (non-specific) with the NKCC2 antibody. Two ladder lanes, one on each side.

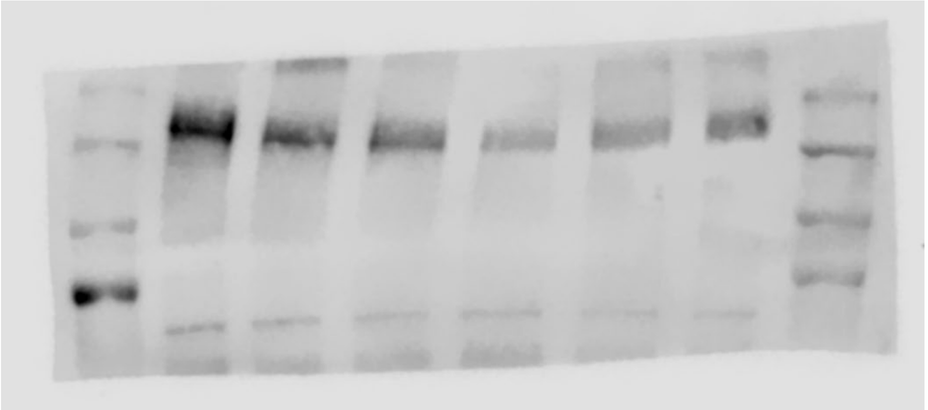

Tubulin (loading control) for 6 week NKCC2 blot – Figure 5M

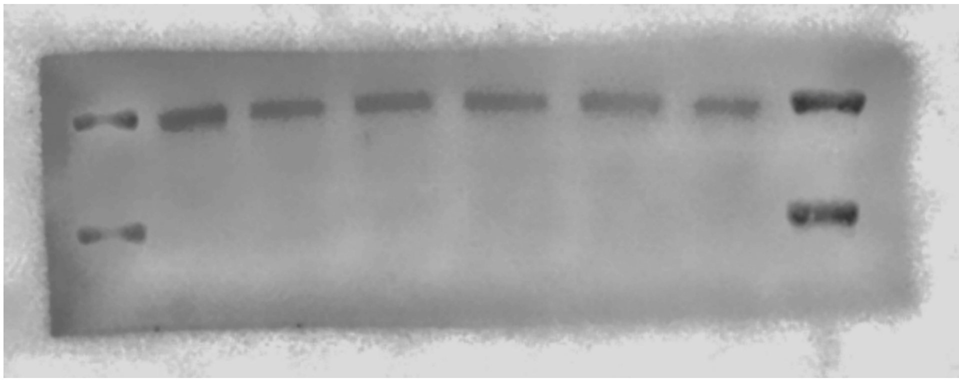

Figure 5N

9 week NKCC2 blot.

Both membrane strips top (probed for NKCC2) and bottom (probed for tubulin) were exposed together in LiCor in this instance. Membrane layout is clearly visible. Consistent bands little above 150kDa ladder bands (predicted mol.wt for NKCC2). Also a consistent band with a varying intensity lower than 75kDa (non-specific) with the NKCC2 antibody. Two ladder lanes to the right, one on the left.

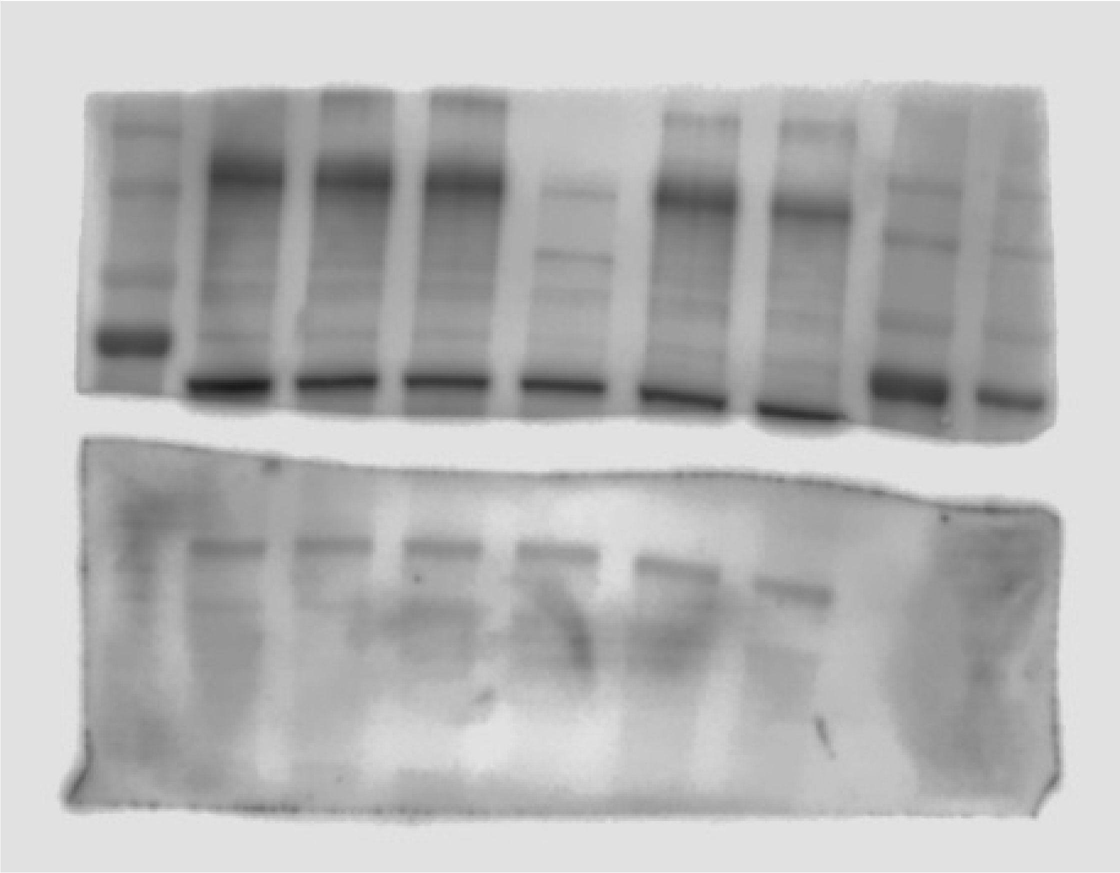

Figure 5O

12 week NKCC2 blot

Both membrane strips top (probed for NKCC2) and bottom (probed for tubulin).
Membrane layout is clearly visible. Two ladder lanes, one on each side.

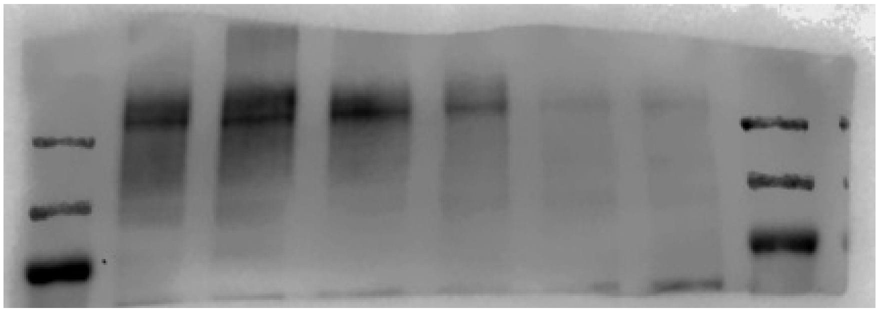

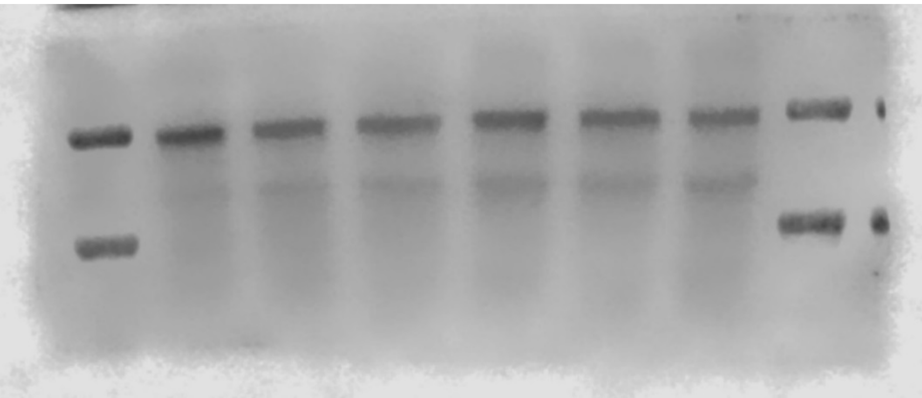

Figure 8A and 8B

9 week and 12 week ATF6 Blots

All western blot images shown in this document and in the main manuscript are all from the chemiluminescence channel in Licor imager. Some ladder bands come through in this channel in most of our blots.

For ATF6 raw data, we are presenting the merged LiCor image from 700nm and chemiluminescence channel to clearly show the ladders and also because there are many non-specific bands for ATF6. The data shown in Figure 8 are the bands running slightly above the 100kDa ladder band. ATF6 predicted molecular weight is 109kDa. Membranes cut into two halves. Top part probed for ATF6 and bottom probed for tubulin.

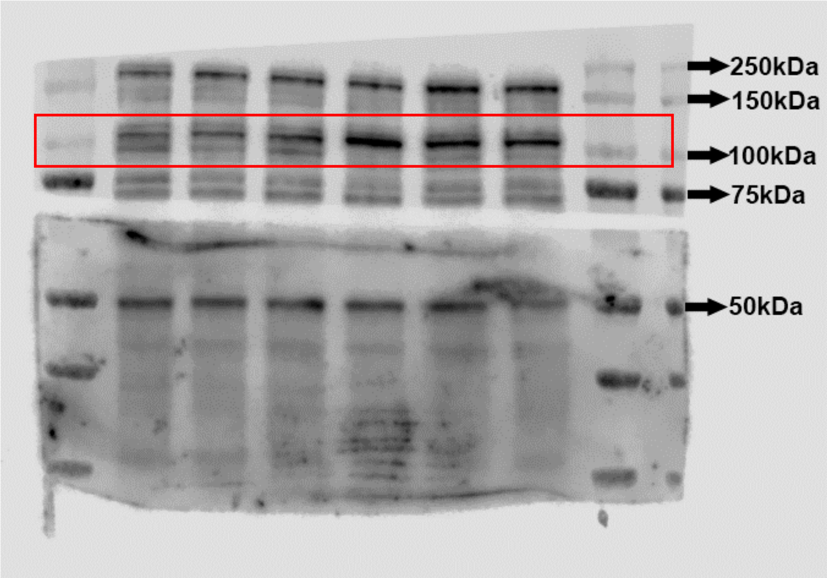

9week – Figure 8A

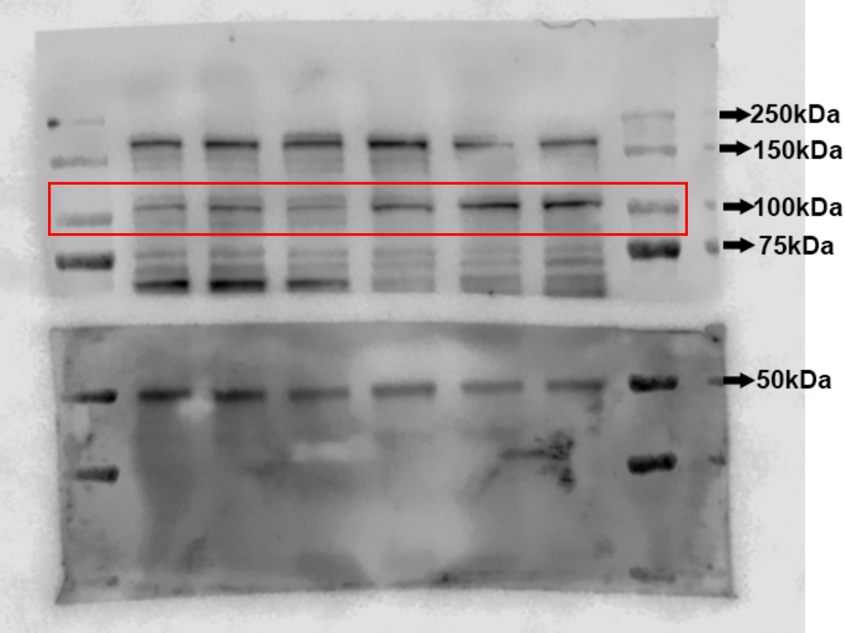

12week – Figure 8B

Figure 8C and 8D

6 week and 12 week XBP1 blots

Both membrane strips top (probed for TFRC) and bottom (probed for XBP1). Membrane layout is clearly visible. Three ladder lanes in 6 week gel, two on the right side. Two ladder lanes in 12 week gel, one on each side.

6 week TFRC (top portion) and XBP1 (bottom portion) – Figure 8C

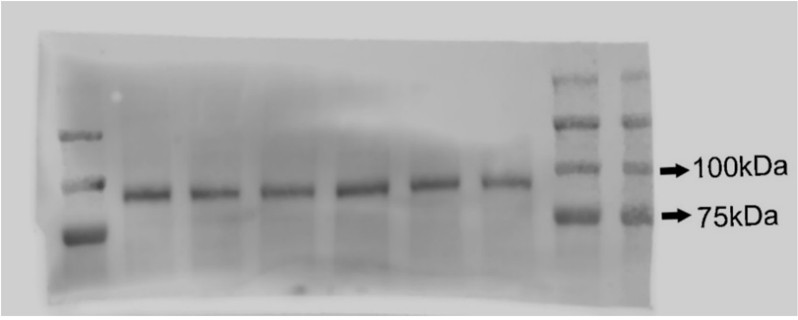

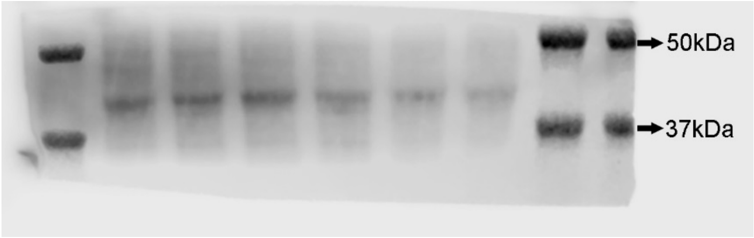

12 week TFRC (top portion of the membrane) - Figure 8D

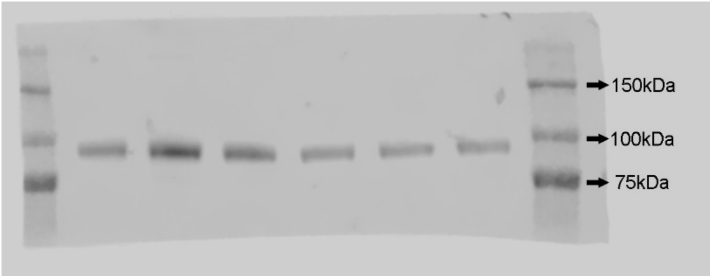

12 week XBP1 (bottom portion of the membrane) – Figure 8D

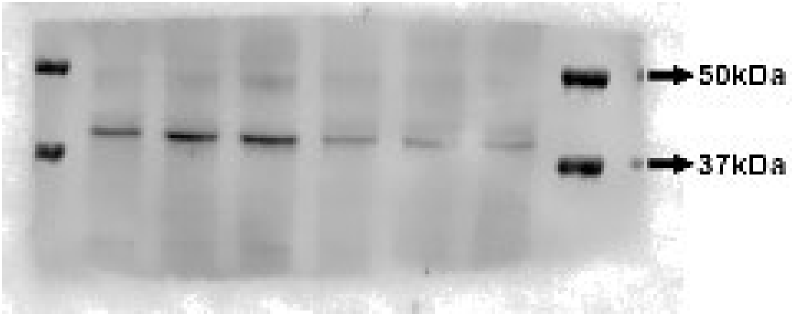

Figure 9E - Both membrane strips top (probed for TFRC) and bottom (probed for Calreticulin) were exposed together in LiCor in this instance. Membrane layout is clearly visible. Three ladder lanes, one ladder lane to left is partially cut off, one ladder lane to the right is mostly cut off in both 9 week and 12 week gels.

9 week Calreticulin blot

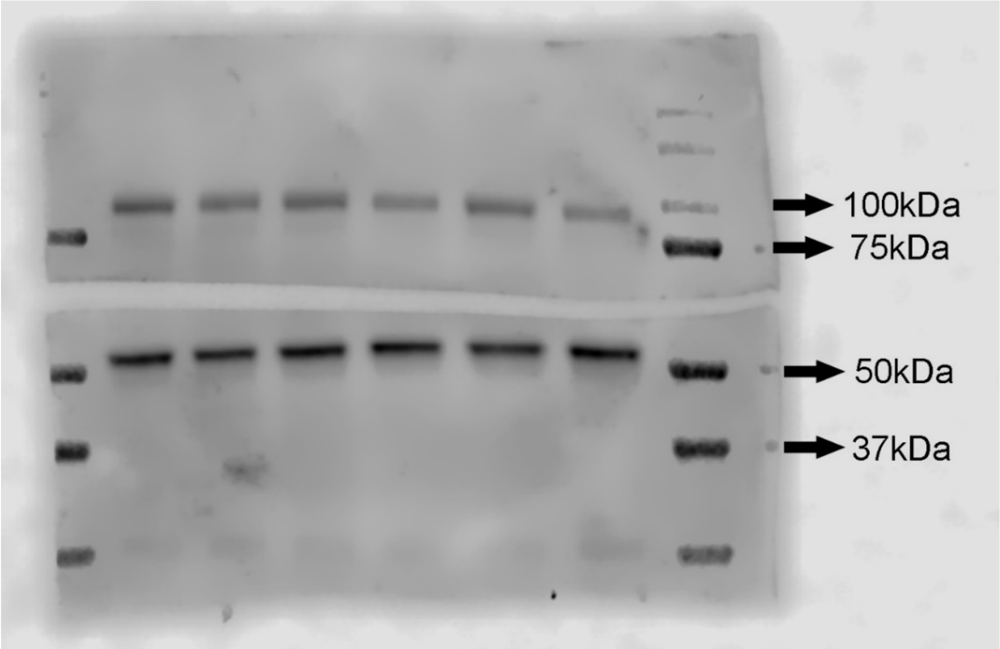

12 week Calreticulin blot

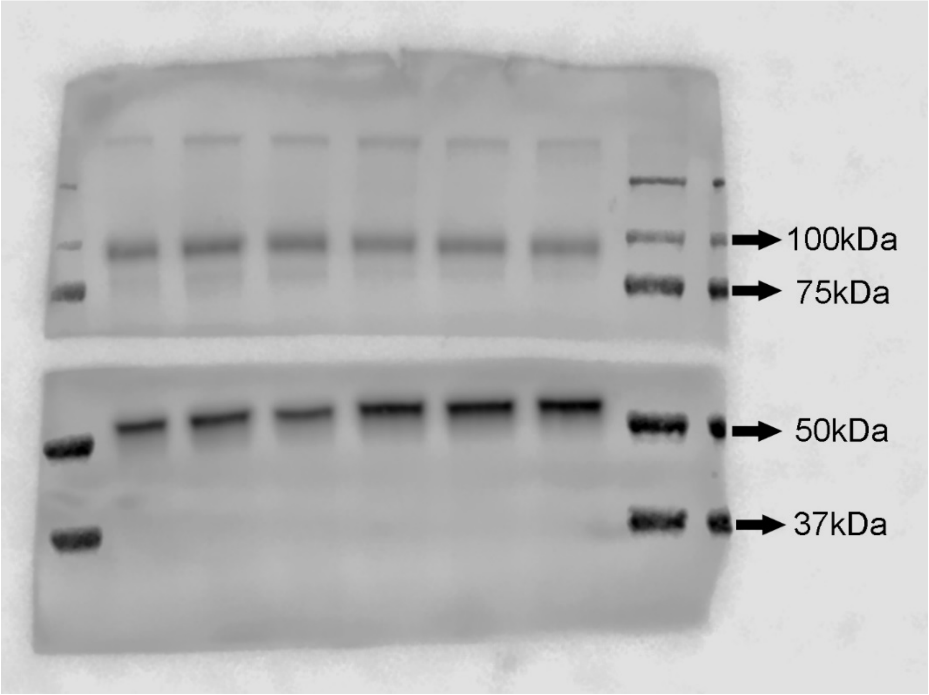

Figure 10E - Both membrane strips top (probed for Calnexin) and bottom (probed for tubulin). Membrane layout is clearly visible. Three ladder lanes, one to the left and two to the right.

9 week Calnexin

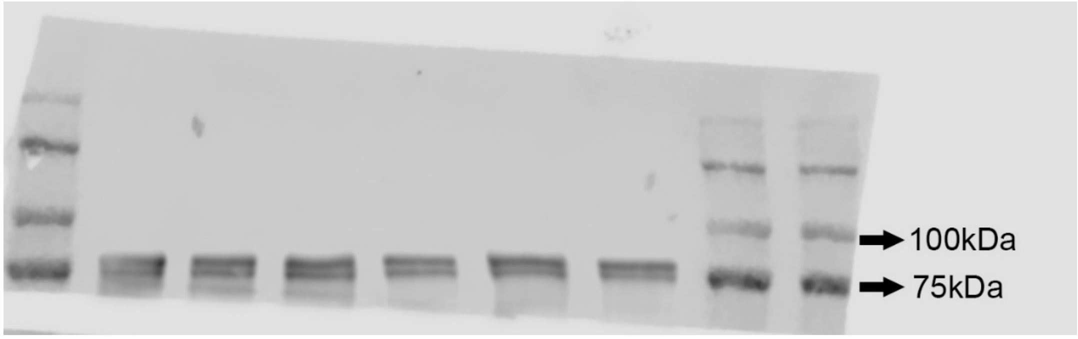

9 week tubulin band (bottom portion of the membrane)

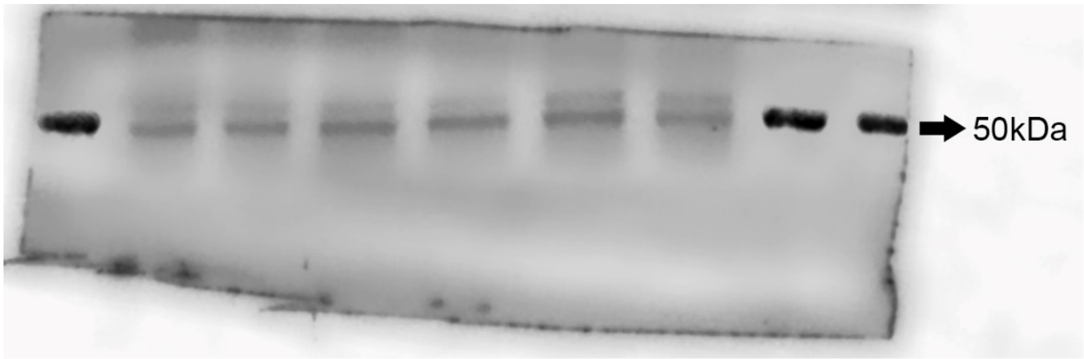

Figure 10E - Both membrane strips top (probed for Calnexin) and bottom (probed for tubulin). Membrane layout is clearly visible. Two ladder lanes, one on each side.

12 week Calnexin

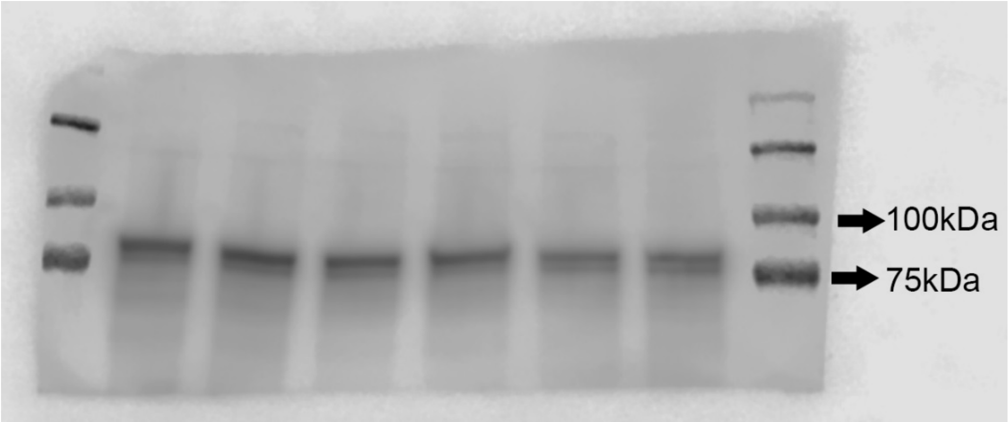

12 week tubulin band (bottom portion of the membrane)

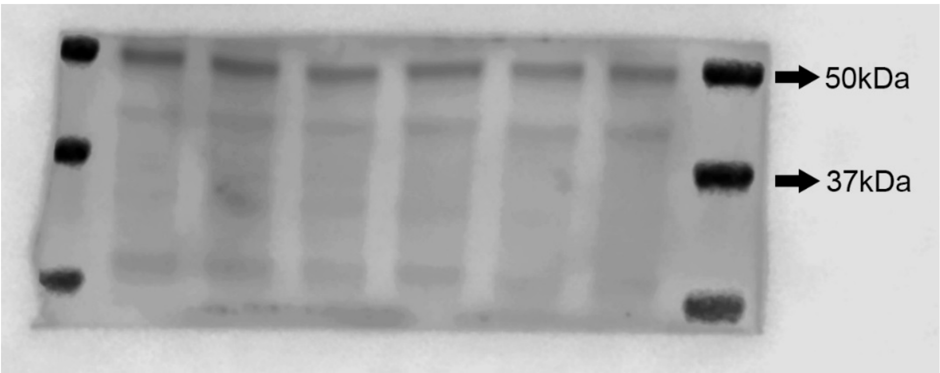

Figure S7

Urinary KIM1 blot: Bands visible between 37 kDa – 75kDa ladders (marked with blue arrows) only in the 3 cKO samples.

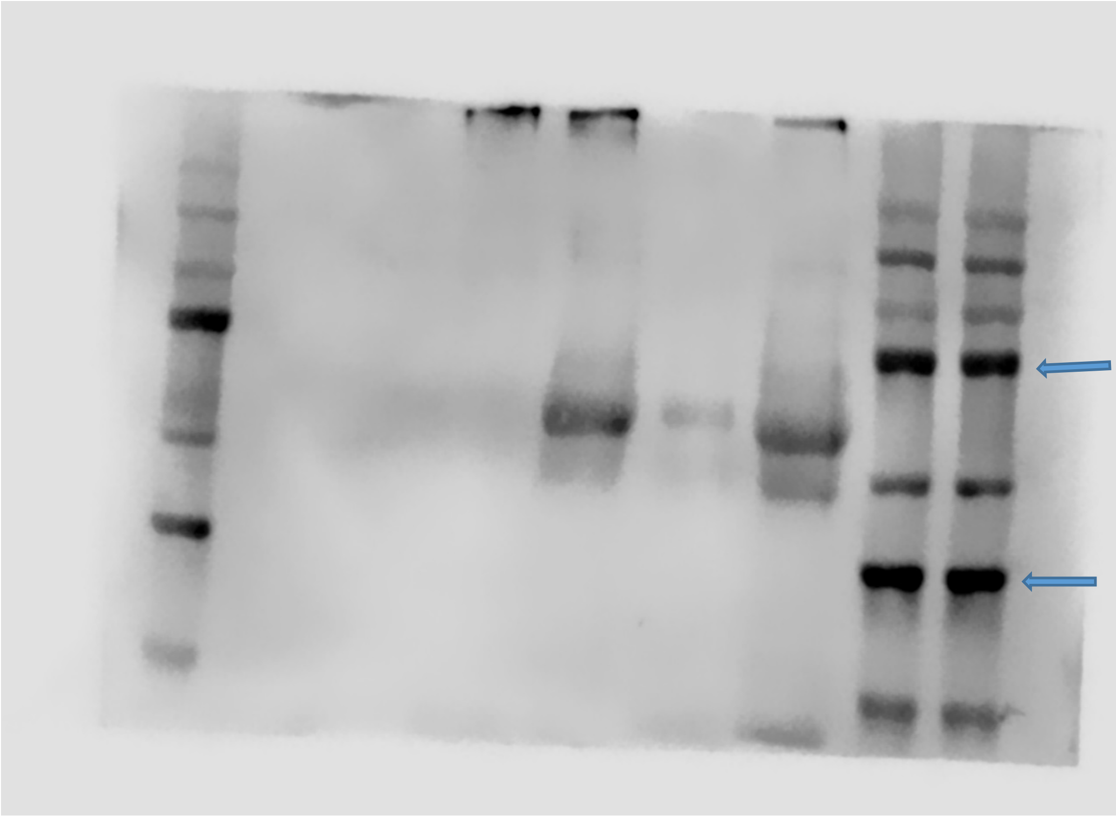

Figure S10

SGLT2 Blot – top probed for SGLT2 and bottom probed for GAPDH.

Three ladder lanes, two on the right side.

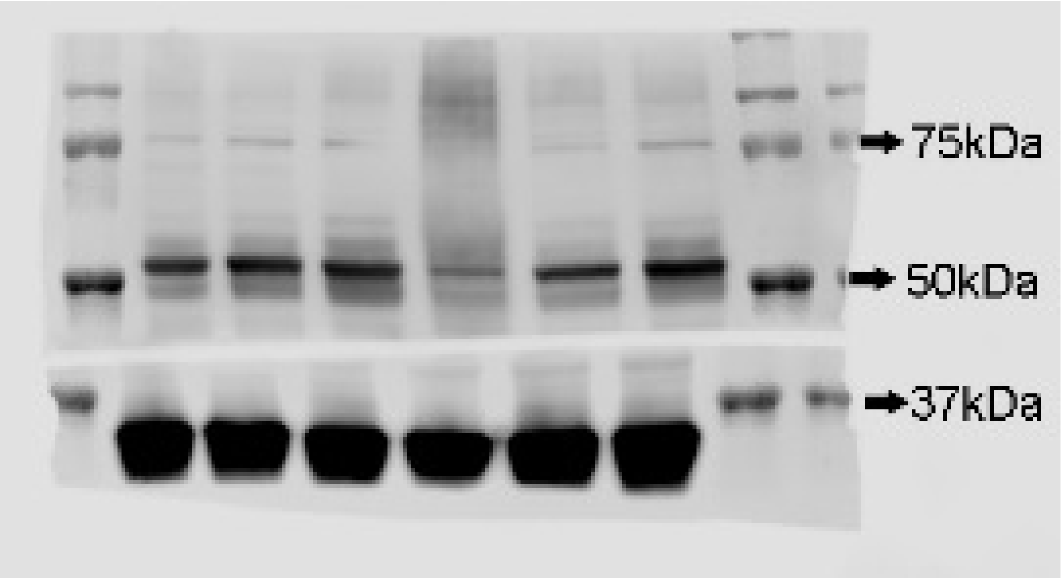

NHE3 blot: Top portion probed for NHE3 and bottom for tubulin.

Three ladder lanes on the gel, one to the left and two to the right with empty lane in the middle.

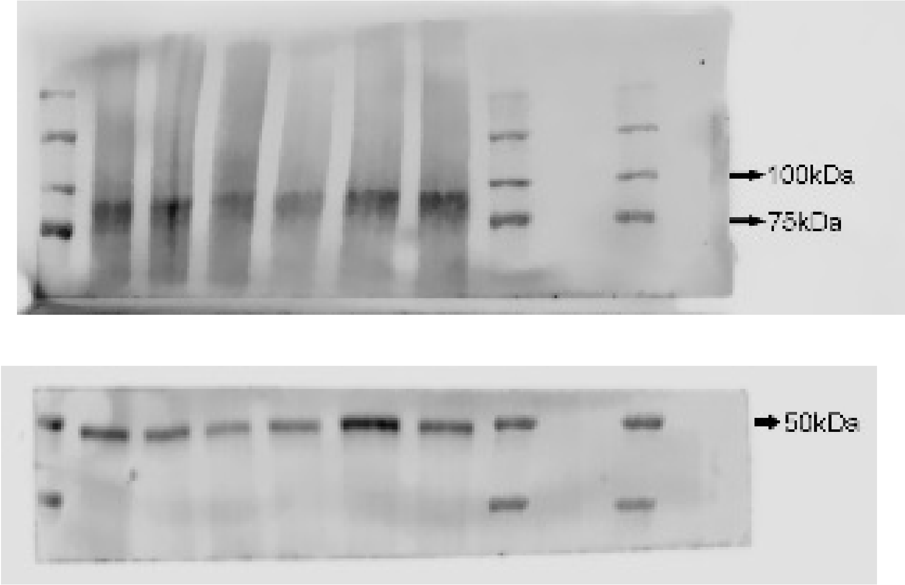

Figure S13

pNCC Blot – top portion probed for pNCC and bottom probed for tubulin.

Three ladder lanes in the gel, one to the left and two to the right.

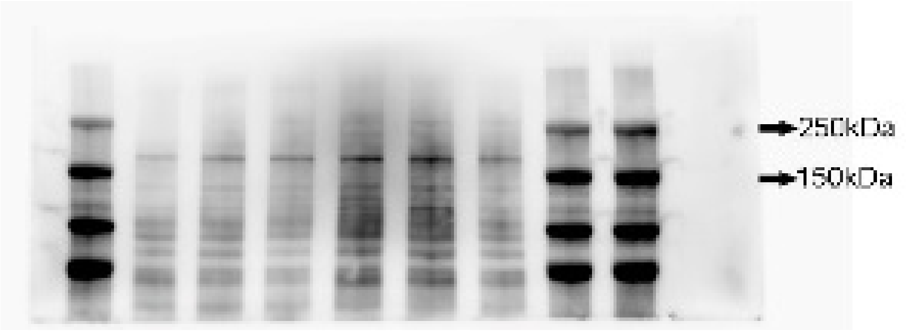

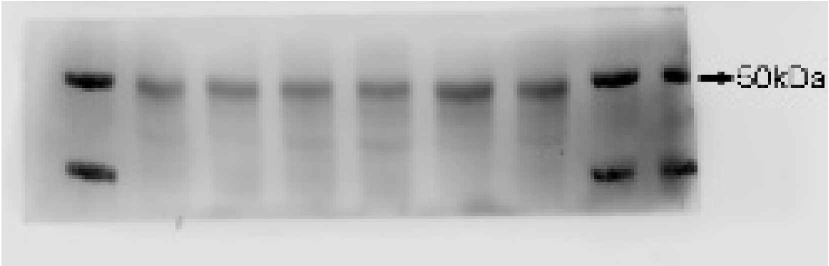

Supplement: supplemental data [file jciinsight-5-138530-s058.pdf]
